# Gas-resolved energy partitioning in a high-frequency sonoreactor: decoupling cavitation-driven reactivity from non-cavitational dissipation

**DOI:** 10.1016/j.ultsonch.2026.107953

**Published:** 2026-07-07

**Authors:** Oualid Hamdaoui, Abdulmajeed Baker, Lahssen El Blidi, Mohamed K. Hadj-Kali

**Affiliations:** Chemical Engineering Department, College of Engineering, King Saud University, 12372 Riyadh, Saudi Arabia

**Keywords:** Sonoreactor, Acoustic cavitation, Gas-resolved energy partitioning, Calorimetry, Cavitation dosimetry, Sonochemiluminescence, Dissolved gas effects

## Abstract

A gas-resolved energetic and mechanistic framework was developed to determine how acoustic power dissipated in a high-frequency sonoreactor is partitioned between cavitation-dependent and non-cavitational contributions, and how this partitioning governs sonochemical reactivity. The approach combines calorimetry with a ΔP-based energetic analysis, using boiled, cooled without headspace, and subsequently vacuum-treated ultrapure water as a cavitation-suppressed reference, together with multimodal cavitation assessment by KI dosimetry, KI-ammonium heptamolybdate (AHM) dosimetry, Fricke dosimetry, H_2_O_2_ dosimetry, 4-nitrophenol (4-NP) dosimetry, sonochemiluminescence, and Sunset Yellow FCF (SSY) degradation under unsparged water and water nominally conditioned with Ar, He, CO_2_, O_2_, and air at 60 and 100 W. Calorimetry showed that a substantial fraction of the total power dissipated in the liquid persisted in the vacuum-treated gas-depleted reference, demonstrating the presence of a dominant non-cavitational baseline, while the gas-dependent excess defined an apparent cavitation contribution. In contrast to the relatively moderate gas effect observed calorimetrically, the chemical and optical probes revealed strong gas-specific pathway selectivity. KI and KI-AHM highlighted interfacial and peroxide-assisted oxidation, Fricke and H_2_O_2_ emphasized bulk oxidizing capacity, 4-NP identified argon as the most effective condition for direct aromatic hydroxylation, and SSY degradation showed that oxygen and argon gave the highest overall oxidative performance. This work introduces an integrated strategy that resolves not only how much power is dissipated in the liquid, but also how dissolved gas composition governs the chemical destiny of that power.

## 1 Introduction

Acoustic cavitation is the fundamental origin of sonochemical reactivity in liquid-phase ultrasound processing, yet the relation between electrical input, power actually dissipated in the liquid, and chemically productive cavitation remains insufficiently resolved in most sonoreactor studies. Bubble oscillation, growth, and collapse generate localized extreme conditions that can produce radicals, oxidants, luminescence, and substrate transformation, but the efficiency with which delivered energy is converted into each of these outcomes depends strongly on reactor hydrodynamics, frequency, power density, and dissolved gas composition [1], [2], [3], [4], [5]. As a result, a sonoreactor can exhibit substantial energy dissipation without necessarily exhibiting the same level of radical production or substrate degradation. This distinction is central to reactor analysis, but it is still often obscured when calorimetry or a single dosimetry method is used in isolation [2], [6].

Calorimetry remains one of the most widely used approaches for quantifying the acoustic power dissipated in a liquid because it directly measures the temperature rise induced by ultrasonication [7], [8], [9]. However, calorimetry is intrinsically a global energetic measurement. It integrates every pathway by which ultrasonic energy is ultimately thermalized in the liquid, including not only cavitation-related dissipation but also viscous losses, acoustic streaming, frictional effects, and bulk heating [1]. Consequently, calorimetry alone cannot identify how much of the measured power is actually associated with chemically productive cavitation. This limitation is especially important in high-frequency systems, where radical generation can be highly sensitive to local bubble dynamics even when the total calorimetric signal changes only modestly [6], [10], [11], [12], [13].

Dissolved gas composition is one of the most influential variables controlling this conversion of energy into chemistry. Gas identity affects bubble nucleation, gas diffusion, thermal conductivity, heat capacity ratio, collapse severity, luminescence, and radical formation [3], [14], [15], [16], [17], [18], [19]. Experiments performed under different gas environments have shown that oxygen, argon, helium, air, and carbon dioxide can produce markedly different sonochemical outcomes, even when the total acoustic input is kept nominally constant [3], [14], [15]. In particular, oxygen-rich conditions can favor oxidant accumulation and bulk oxidizing capacity, whereas argon often promotes chemically aggressive collapse events, and carbon dioxide commonly suppresses the most active cavitation regimes [3], [14], [15]. These gas-specific effects imply that the same apparent acoustic power can be translated into very different chemical outputs depending on the dissolved gas environment.

A second limitation in current reactor characterization is the tendency to rely on a single probe to describe cavitation activity. KI dosimetry, KI with ammonium heptamolybdate (AHM), Fricke dosimetry, hydrogen peroxide formation, aromatic hydroxylation probes such as 4-nitrophenol (4-NP), and luminescence-based methods do not interrogate the same radical or oxidant pathway [6], [10], [20], [21]. KI reflects interfacial oxidation, KI-AHM extends the analysis toward peroxide-assisted oxidation, Fricke reports a broader oxidizing inventory, hydrogen peroxide reports the recombination branch, and 4-NP reflects direct aromatic hydroxylation [6], [10], [20], [21]. Sonochemiluminescence (SCL) adds a spatially resolved optical perspective on active cavitation zones, while substrate degradation integrates the practical consequence of these parallel pathways [14], [22]. No single method can therefore represent the full chemical meaning of cavitation. A mechanistic interpretation of sonoreactor performance requires a framework that links global energetics to multiple orthogonal cavitation probes.

This divergence arises because calorimetry and dosimetry do not sample the same physical quantity. Calorimetry measures the final thermalized outcome of acoustic energy dissipation in the liquid, whereas chemical and optical probes sample specific products or locations within the cavitation field. Gas identity modifies the bubble interior through changes in solubility, compression thermodynamics, heat leakage, and gas-phase chemistry. These changes determine whether the gas-enabled energy is expressed as interfacial radical attack, peroxide accumulation, bulk oxidizing capacity, luminescence, or substrate degradation. Therefore, an energetic partition such as ΔP must be interpreted together with probe-specific chemical selectivity rather than as a universal cavitation-intensity number [3], [23].

Recent work has begun to show the value of combining calorimetry with complementary dosimetries in high-frequency sonoreactors [10]. Nevertheless, a major gap remains: the energetic signal measured calorimetrically is still rarely partitioned into a cavitation-dependent contribution and a non-cavitational contribution in a way that can be compared directly with orthogonal dosimetry and degradation data. This gap is particularly important when dissolved gas composition is intentionally varied, because gas effects may alter not only radical generation but also the relative magnitude of background dissipation that persists even when chemically productive cavitation is strongly reduced [3], [6], [14]. A rigorous interpretation therefore requires an energetic framework in which calorimetry defines the total power dissipated in the liquid, while the gas-dependent excess over a cavitation-suppressed reference is used to estimate the apparent cavitation contribution.

In this work, a gas-resolved energetic and mechanistic framework is developed for a high-frequency sonoreactor operated in the vacuum-treated gas-depleted reference, in unsparged water, and in water nominally conditioned with Ar, He, CO_2_, O_2_, and air at two electrical powers. The total acoustic power dissipated in the liquid is first determined calorimetrically and then interpreted using a ΔP-based energetic partitioning framework, in which the vacuum-treated gas-depleted reference is used as the cavitation-suppressed reference to distinguish an apparent cavitation-dependent contribution from a non-cavitational baseline. This energetic analysis is subsequently linked to a multimodal cavitation assessment combining KI dosimetry, KI-AHM dosimetry, Fricke dosimetry, H_2_O_2_ dosimetry, 4-NP dosimetry, SCL, and Sunset Yellow FCF degradation.

**Fig. 1:**
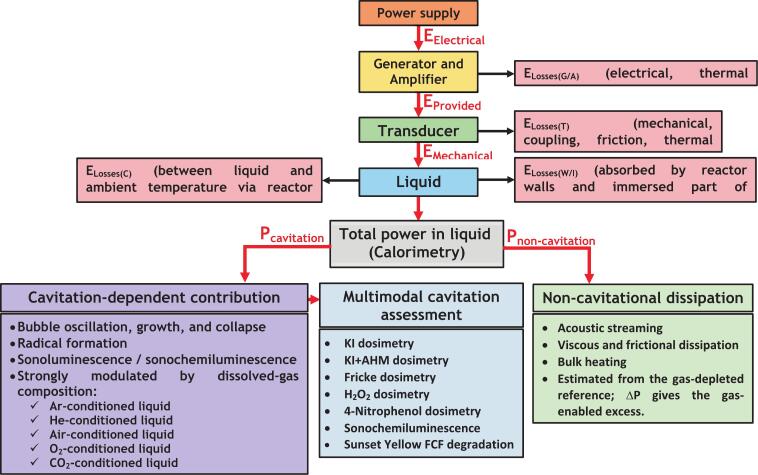
Conceptual framework illustrating the gas-resolved energetic partitioning of total acoustic power dissipated in a sonoreactor into cavitation-dependent and non-cavitational contributions, and its connection with multimodal cavitation assessment.

Fig. 1 summarizes the conceptual basis of this study. The scheme distinguishes the total power dissipated in the liquid, obtained by calorimetry, from its two operational components, namely the cavitation-dependent contribution and the non-cavitational dissipation. It further links the cavitation-dependent contribution to a multimodal assessment based on oxidative dosimetry, optical cavitation imaging, and substrate degradation. This framework is designed to answer a question that remains insufficiently addressed in sonochemical reactor analysis: not only how much power is dissipated in the liquid, but also how that power is partitioned between background dissipation and chemically productive cavitation, and how dissolved gas composition governs that partitioning.

Accordingly, the novelty of the present work is not the individual use of calorimetry or dosimetry, but their integration within a gas-resolved ΔP-based energetic partitioning framework that distinguishes a non-cavitational baseline from an apparent cavitation contribution and then maps this contribution across multiple oxidative pathways. This strategy makes it possible to determine not only how much power is dissipated in the liquid, but also how dissolved gas composition governs the chemical destiny of that power. By coupling calorimetry with complementary chemical and optical probes, the study provides a more discriminating basis for reactor analysis than calorimetry or dosimetry alone and offers a transferable strategy for interpreting gas effects in high-frequency sonoreactors.

## 2 Material and methods

### 2.1 Chemicals

Sunset Yellow FCF (SSY), potassium iodide, ammonium heptamolybdate (AHM), sulfuric acid, ferrous ammonium sulfate, sodium chloride, 4-nitrophenol (4-NP), sodium hydroxide, luminol, and all other reagents were of analytical grade and were used as received. Solutions were prepared with Milli-Q water.

### 2.2 Sonoreactor and operating conditions

Experiments were carried out in the same 425 kHz sonoreactor configuration described by Baker et al. [10], [24], consisting of a jacketed cylindrical glass vessel equipped with a bottom-mounted piezoelectric transducer. The reactor was operated at two electrical input powers, 60 and 100 W. A constant liquid volume of 200 mL was used in all experiments. Temperature was controlled at 20 °C by external recirculation through the reactor jacket, except during calorimetric runs, for which the jacket was emptied as described below.

### 2.3 Gas conditioning and vacuum treatment

Three liquid preparation modes were investigated: unsparged water, vacuum-treated gas-depleted water, and water nominally conditioned with Ar, He, CO_2_, O_2_, or air. Unsparged experiments were performed without intentional gas bubbling prior to ultrasonication. Gas-conditioned solutions were prepared by bubbling the selected gas through the liquid for 15 min immediately before ultrasound application. The term “gas-conditioned” is used here as an operational descriptor of this standardized preparation protocol; it does not imply that the dissolved concentration of each gas was directly measured before ultrasonication. Dissolved-gas concentrations before and after ultrasonication were not measured in this study. No membrane-inlet mass spectrometry, gas chromatography, or dissolved-gas-specific probe was used to quantify Ar, He, CO_2_, O_2_, or the individual components of air. Therefore, the gas variable in this work should be interpreted as a controlled preparation condition rather than as an independently measured dissolved-gas concentration. Literature Henry's-law solubility trends and thermophysical properties are used only to rationalize the expected relative behavior of the gases, not to assign experimental gas concentrations. Vacuum-treated solutions were prepared using a two-step degassing protocol. Ultrapure water was first boiled to remove a large fraction of dissolved gases and then cooled to room temperature in a completely filled sealed vessel without headspace, in order to minimize gas re-equilibration during cooling. Immediately before ultrasonication, the cooled degassed water was transferred carefully to the reactor and further degassed under reduced pressure for 20 min. The absolute pressure during vacuum treatment was < 1 kPa. This procedure was used to minimize the dissolved-gas inventory and suppress gas-assisted cavitation before the calorimetric, dosimetric, SCL, and degradation measurements, which were then performed using the gas-depleted liquid without continuous pumping during irradiation. Because boiling followed by headspace-free cooling and vacuum treatment cannot prove the complete elimination of all microscopic gas nuclei, this condition is referred to as a cavitation-suppressed or cavitation-minimized reference rather than an absolutely cavitation-free state. After vacuum pretreatment, the vacuum-treated liquid was used for ultrasonication under the same reactor configuration, liquid volume, and temperature conditions as the other gas-conditioned experiments. No continuous evacuation was applied during ultrasound irradiation; therefore, the term “vacuum” denotes the prior liquid degassing step and the resulting gas-depleted reference state, not ultrasonication under sustained reduced pressure. Similarly, the gas-conditioned experiments were compared on the basis of identical pre-bubbling protocols rather than measured dissolved-gas concentrations during irradiation.

The pH was measured after gas conditioning and after ultrasonication. CO_2_-conditioned solutions showed the largest pH decrease; however, pH-matched controls prepared by adjusting non-CO_2_ solutions to the same pH range showed no statistically significant effect on KI, KI-AHM, or SSY responses. Therefore, the influence of gas-induced pH modification was considered negligible under the present conditions.

### 2.4 Calorimetric determination of dissipated power

The acoustic power dissipated in the liquid was determined by the temperature rise calorimetric method. For each condition, the initial rate of temperature increase during the first 5 min of irradiation was measured and the calorimetric power was calculated. During calorimetric measurements, Milli-Q water was used and the cooling jacket was left empty to minimize external heat exchange. Matrix-specific calorimetry was not performed for each dosimetric solution. Thus, the calorimetric values reported here represent a standardized water-based acoustic reference measured under the same volume, gas-conditioning, temperature, and electrical-power conditions used for the chemical experiments. The possible matrix bias was considered through the calorimetric relation P_cal_ = ρVC_p_(dT/dt). Differences between Milli-Q water and the aqueous probe matrices may affect ρC_p_ and the temperature-rise slope. Therefore, the ΔP values are interpreted as standardized water-based energetic references rather than matrix-specific acoustic powers. This method was used because calorimetry is the accepted reference approach for estimating the acoustic power absorbed and dissipated in the liquid phase.

### 2.5 KI and KI-AHM dosimetry

KI dosimetry was performed using a 0.10 M KI solution under the selected gas and power conditions. Cavitation-generated oxidizing species converted iodide into iodine, which rapidly formed triiodide. The triiodide concentration was measured spectrophotometrically at 351 nm in a 1 cm quartz cuvette using the same extinction coefficient and analytical procedure adopted by Baker et al. [10]. The dosimetric response was expressed as the slope of triiodide concentration versus ultrasonication time.

For the KI-AHM assay, the same iodometric platform was used in the presence of AHM, which catalyzes oxidation of iodide by hydrogen peroxide formed during cavitation. The difference between the catalyzed and uncatalyzed responses was taken as an indicator of the peroxide-assisted oxidation branch, in accordance with the protocol and interpretation reported by Baker et al. [10].

pH-matched controls confirmed that the pH shift induced by CO_2_ conditioning did not significantly affect the KI or KI-AHM response.

### 2.6 Hydrogen peroxide dosimetry

Hydrogen peroxide formation was quantified iodometrically. At selected irradiation times, aliquots were withdrawn and reacted with KI and AHM, then the absorbance of the resulting triiodide was measured at 351 nm. H_2_O_2_ concentrations were calculated from the Beer-Lambert law and the formation rates were determined from the slopes of concentration versus time.

### 2.7 Fricke dosimetry

Fricke dosimetry was carried out using an acidic solution containing 0.4 M H_2_SO_4_, 1.0 mM ferrous ammonium sulfate, and 1.0 mM NaCl. Oxidizing species formed during cavitation converted Fe^2+^ into Fe^3+^, and the ferric ion concentration was measured spectrophotometrically at 303 nm in a 1 cm quartz cuvette. The Fe^3+^ production rate was obtained from the slope of concentration versus time.

### 2.8 4-nitrophenol dosimetry

Hydroxyl radical attack on an aromatic substrate was evaluated using 4-NP dosimetry. A 200 mL solution of 4-NP at 1.0 mM was adjusted to pH 5.0 and irradiated under the selected conditions. At defined intervals, aliquots were withdrawn and mixed with 0.20 M NaOH to convert 4-nitrocatechol (4-NC) into its strongly absorbing phenolate form. The absorbance was measured at 510 nm, and the 4-NC formation rate was determined from the slope of concentration versus time.

### 2.9 Sonochemiluminescence imaging

SCL was recorded using the same luminol system and image acquisition conditions described by Baker et al. [25]. A 1 mM luminol solution prepared in 0.1 M NaOH was irradiated under the selected gas, power, temperature, and volume conditions. Images were acquired with a Nikon DSLR camera equipped with an 18 to 55 mm zoom lens, using exposure times of 30 and 60 s to resolve the spatial distribution of cavitation active zones. The measurements were performed in a dark environment and were used qualitatively to compare the relative intensity and spatial extent of cavitation-related light emission among gas conditions.

### 2.10 SSY degradation experiments

SSY degradation experiments were carried out under the same ultrasonication, temperature, volume, and gas conditioning conditions as the dosimetry tests. The dye concentration was monitored spectrophotometrically at 482 nm. pH-matched controls confirmed that the pH shift induced by CO_2_ conditioning did not significantly affect SSY absorbance at 482 nm or the degradation profile. Aliquots were withdrawn at selected times, analyzed immediately, and returned to the reactor so that the working volume remained constant.

### 2.11 Replication and statistical analysis

Each experiment was carried out in triplicate. Reported values were expressed as the mean of three independent runs. Differences between conditions were evaluated using two-tailed Student’s t-tests, and statistical significance was taken at *p* < 0.03.

The same statistical criterion was used to evaluate pH-matched controls. pH effects were considered negligible when no statistically significant difference was observed relative to the corresponding unadjusted control.

## 3 Results and discussion

### 3.1 Calorimetric determination of total power in liquid

**Fig. 2:**
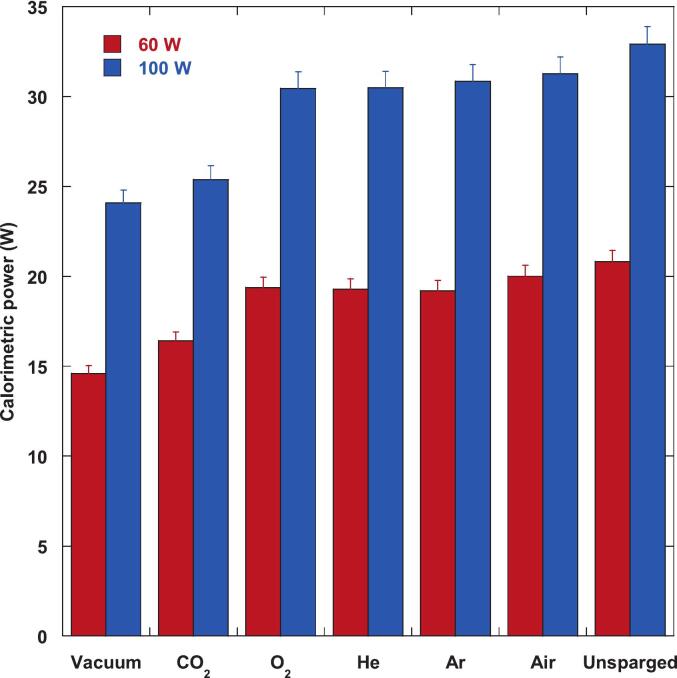
Calorimetric power measured in the vacuum-treated gas-depleted reference, unsparged, and gas-conditioned media at 60 and 100 W (volume: 200 mL, frequency: 425 kHz).

Fig. 2 shows that the calorimetric power increased systematically when the electrical input was raised from 60 to 100 W under all gas conditions, but the influence of dissolved gas remained modest compared with the much larger differences later observed in the chemical probes. At 60 W, the calorimetric power was approximately 14.6 W in the vacuum-treated gas-depleted reference, 16.4 W in CO_2_-conditioned water, 19.2 to 19.4 W in Ar-, He-, and O_2_-conditioned water, 20.0 W in air-conditioned water, and 20.8 W in unsparged water. At 100 W, the corresponding values increased to about 24.1 W, 25.4 W, 30.4 to 30.9 W, 31.3 W, and 32.9 W, respectively. This relatively compressed ranking is mechanistically important because calorimetry measures the total acoustic power dissipated in the liquid through the temperature rise and does not selectively report cavitation chemistry [2], [10]. In other words, calorimetry integrates all dissipative channels that are ultimately thermalized in the liquid, including both cavitation-related dissipation and non-cavitational contributions such as acoustic streaming, viscous friction, and bulk hydrodynamic losses [2], [4]. Because calorimetry was performed in Milli-Q water, the resulting P_cal_ values should be interpreted as common energetic reference values for the reactor rather than matrix-specific calorimetric powers for KI, KI-AHM, Fricke, luminol, 4-NP, or SSY solutions. Matrix-dependent changes in density, heat capacity, viscosity, ionic strength, and acoustic attenuation may introduce small systematic deviations. However, the ΔP analysis was applied consistently to compare gas-conditioning states under a fixed reference matrix, and the much larger differences observed among the dosimetric responses are interpreted as gas-dependent cavitation-chemistry effects rather than as calorimetric matrix artifacts.

A more rigorous interpretation of this gas-dependent but relatively compressed calorimetric ranking requires consideration of the thermophysical properties of the dissolved gases. Gas identity affects cavitation through at least four coupled physical descriptors: solubility in water, heat capacity ratio γ = C_p_/C_v_, gas thermal conductivity/thermal diffusivity, and chemical participation inside the bubble [3], [26]. Solubility controls the dissolved-gas inventory available for bubble nucleation and rectified diffusion. The heat capacity ratio controls the temperature rise expected during rapid compression, with higher γ favoring stronger adiabatic heating [3], [26]. Thermal conductivity and diffusivity determine the extent of heat leakage from the compressed bubble interior to the surrounding liquid during collapse, thereby acting as a thermal-shielding or thermal-leakage pathway [3], [26]. Finally, the number and spatial density of bubbles affect acoustic attenuation: dense or highly stable bubble populations can scatter, absorb, and shield the acoustic field, reducing the pressure amplitude available for violent collapse while still converting acoustic energy into heat [3]. These thermophysical arguments are based on literature gas-property and Henry-law solubility trends, not on direct measurements of dissolved-gas concentrations in the present experiments. The gas-conditioned cases are therefore interpreted as reproducible initial preparation states established by identical bubbling times, liquid volume, temperature, and reactor geometry. Because cavitation itself can modify dissolved-gas content through bubble growth, gas exchange, and local heating, the post-sonication dissolved-gas concentrations were not assumed to remain equal to equilibrium saturation values. The analysis therefore avoids using absolute gas concentration as an input parameter and instead compares the calorimetric, dosimetric, optical, and degradation responses obtained under standardized gas-conditioning protocols.

The physical meaning of the vacuum-treated reference must be interpreted in light of the degassing protocol used before ultrasonication. The vacuum-treated liquid was not obtained by headspace evacuation alone. Ultrapure water was first boiled to strip dissolved gases, then cooled in a completely filled sealed vessel without headspace to limit atmospheric gas reabsorption, and finally subjected to vacuum treatment at < 1 kPa for 20 min immediately before use. This combined thermal/headspace-free/vacuum protocol was designed to minimize the dissolved-gas inventory and to strongly suppress gas-assisted cavitation. Nevertheless, it is not interpreted as proof that all microscopic gas nuclei were eliminated. For this reason, the vacuum-treated gas-depleted reference is treated as an experimentally accessible cavitation-suppressed reference, and the calorimetric excess over this reference is defined as an apparent cavitation-dependent contribution.

A first major mechanistic insight from Fig. 2 is that a substantial calorimetric signal persists even in the vacuum-treated gas-depleted reference. This condition gave 14.6 W at 60 W input and 24.1 W at 100 W input, corresponding to 70.2% and 73.3% of the total calorimetric signal measured in unsparged water at the same electrical powers. Since degassing strongly suppresses bubble nucleation and cavitation activity, this remaining thermal signal cannot be assigned primarily to chemically productive cavitation. It instead defines a lower bound for the non-cavitational baseline of the reactor, arising from processes such as viscous attenuation, oscillatory bulk losses, acoustic streaming, and heat transfer to the liquid and vessel [2], [3]. This interpretation is fully consistent with the cavitation literature, which shows that dissolved gas is essential for efficient cavitation development, whereas calorimetry remains a global measure of power dissipation rather than a direct measure of radical production [2], [3].

This physical picture leads directly to the ΔP framework. In the present system, the total calorimetric power under each gas condition may be written as the sum of a non-cavitational baseline and an apparent cavitation-dependent contribution, with the latter operationally defined as the excess over the boiled/headspace-free/vacuum-treated reference:

ΔP = P_cal,gas_ − P_cal,vac_.

Under this formulation, P_cal,vac_ provides a lower-bound estimate of the non-cavitational contribution, while ΔP represents the gas-enabled increment that appears when dissolved-gas-assisted cavitation is restored. This gas-enabled increment is not controlled by gas amount alone, but by the combined influence of gas solubility, compression thermodynamics, thermal leakage, and bubble-cloud attenuation [3], [26]. The term apparent is essential here. Although boiling, cooling without headspace, and subsequent vacuum treatment at < 1 kPa strongly reduce the dissolved-gas inventory, this protocol cannot prove the absolute elimination of all microscopic nuclei. The ΔP method therefore does not claim an absolute thermodynamic partition between cavitational and non-cavitational power. Instead, it provides an operational energetic partition based on a deliberately gas-depleted, cavitation-suppressed reference that can be confronted with dosimetry, SCL, and degradation data in a physically meaningful way [2], [10].

Application of this ΔP analysis yields a clear quantitative result. At 60 W, the apparent cavitation contribution was approximately 1.8 W for carbon dioxide, 4.6 W for argon, 4.7 W for helium, 4.8 W for oxygen, 5.4 W for air, and 6.2 W for unsparged water. At 100 W, these values became about 1.3 W, 6.8 W, 6.4 W, 6.3 W, 7.2 W, and 8.8 W, respectively. Expressed as a fraction of the total calorimetric signal, the apparent cavitation contribution represented only about 11.0% in carbon dioxide, 24.0% in argon, 24.4% in helium, 24.7% in oxygen, 27.0% in air, and 29.8% in unsparged water at 60 W. At 100 W, these fractions were approximately 5.1%, 22.0%, 21.0%, 20.7%, 23.0%, and 26.7%, respectively. These numbers show that the calorimetric response remained dominated by the non-cavitational baseline even in gas-containing media. Thus, calorimetry alone cannot be interpreted as a direct surrogate for cavitation intensity, because most of the thermal signal persists independently of the chemically productive cavitation reflected later in dosimetry [2], [10].

The numerical ΔP values should therefore be interpreted as net energetic outcomes rather than as direct measures of identical cavitation events. For a given electrical input, a gas can increase the calorimetric power by increasing bubble number density, stable oscillation, viscous damping, acoustic attenuation, or collapse intensity [3]. These mechanisms all end as heat in calorimetry, but they do not produce the same sonochemical consequences. Consequently, the gas-dependent calorimetric ranking reflects thermophysical compensation: high γ favors compression heating, high thermal conductivity promotes heat leakage, high solubility modifies bubble population and shielding, and reactive gases modify the chemical fate of collapse products [3], [26]. This explains why several gases can be calorimetrically grouped within a narrow range while later showing strongly divergent KI, Fricke, H_2_O_2_, 4-NP, SCL, and SSY responses.

The low position of carbon dioxide is particularly instructive because it can be rationalized from its coupled solubility and thermodynamic properties rather than from solubility alone. Based on Henry-law solubility data, CO_2_ is expected to be much more soluble in water than He, Ar, O_2_, or the major components of air, and therefore can supply a larger nominal dissolved-gas inventory to the acoustic field [26]. This statement is used as a literature-supported solubility rationale and should not be interpreted as a measured dissolved CO_2_ concentration before or after ultrasonication. However, high gas availability does not necessarily produce violent cavitation. As a polyatomic gas with a relatively low heat capacity ratio, CO_2_ stores a larger fraction of compression work in internal degrees of freedom and produces a lower temperature rise during rapid bubble compression than monatomic gases [3], [26]. In addition, CO_2_-rich bubbles are more susceptible to damping and cushioning, while the associated bubble population can enhance scattering, acoustic attenuation, and bubble-cloud shielding [3]. Under these conditions, part of the acoustic energy is dissipated through stable oscillation, viscous damping, and weakly productive thermalization rather than through intense inertial collapse. This explains why CO_2_ increased the calorimetric signal only slightly above the boiled/headspace-free/vacuum-treated reference, by about 1.8 W at 60 W and 1.3 W at 100 W, and gave the smallest apparent cavitation contribution among all gas-conditioned media. The CO_2_ result therefore does not indicate an absence of acoustic energy dissipation; rather, it indicates that the gas-enabled dissipation is diverted toward damped, weakly productive pathways instead of chemically aggressive collapse.

The narrow calorimetric grouping of oxygen, helium, argon, and air can also be understood from the fact that calorimetry integrates several compensating gas-dependent mechanisms. Argon has a high heat capacity ratio and low thermal conductivity, which favor stronger compression heating and more intense collapse events [3]. Helium also has a high heat capacity ratio, but its very high thermal conductivity and diffusivity promote rapid heat leakage from the bubble interior during compression; therefore, helium bubbles can dissipate acoustic energy efficiently as heat without sustaining the same hot-spot severity or radical productivity as argon [3]. Oxygen and air have lower heat capacity ratios than noble gases and intermediate thermal conductivities, but they support chemically reactive bubble contents and stable active bubble populations [3]. These opposing effects compress the calorimetric differences among Ar, He, O_2_, and air into a narrow window, even though the corresponding bubble-collapse chemistry differs substantially. Therefore, the similarity of their calorimetric powers should not be interpreted as evidence for identical cavitation dynamics. It instead reflects the fact that different combinations of compression heating, heat leakage, viscous damping, acoustic streaming, acoustic scattering, and bubble-cloud attenuation can yield similar final heat inputs to the liquid [3].

The highest calorimetric values in unsparged water are especially revealing. Unsparged water gave about 20.8 W at 60 W input and 32.9 W at 100 W input, exceeding all deliberately gas-conditioned media. The apparent cavitation contribution under this condition was also the largest, with ΔP values of about 6.2 and 8.8 W at 60 and 100 W, respectively. This behavior can be attributed to the naturally equilibrated gas mixture and native nuclei present in unsparged water. Unlike single-gas-conditioned media, unsparged water contains a mixed gas inventory and a heterogeneous population of pre-existing nuclei, which can favor broad acoustic coupling, distributed bubble activity, and efficient global thermalization [3]. However, this does not imply that unsparged water produces the most intense collapse events per bubble or the highest sonochemical yield. Rather, it indicates that the unsparged medium is efficient at converting acoustic energy into total heat through a combination of cavitation-related and non-cavitational pathways. The later dosimetry, SCL, and SSY degradation results show that this calorimetric maximum is chemically non-universal: Ar and O_2_ can be more efficient for specific radical or oxidative pathways even when their total calorimetric power is lower than that of unsparged water. Thus, the unsparged-water result reinforces the need to distinguish total acoustic power dissipation from chemically effective cavitation.

The gas-dependent calorimetric ranking is therefore best interpreted as the net result of thermophysical compensation rather than as a direct ranking of collapse intensity. Gas solubility affects bubble availability and acoustic shielding, γ affects compression heating, thermal conductivity affects heat leakage, and gas-phase chemistry affects the fate of hot-spot products [3], [26]. Because these factors act simultaneously, calorimetry yields relatively narrow energetic groupings for several gases, whereas the chemical probes resolve much larger differences in productive cavitation pathways.

A second quantitative insight from Fig. 2 concerns the effect of increasing electrical power. The calorimetric signal increased by about 9.5 W in the vacuum-treated gas-depleted reference, 9.0 W in carbon dioxide, 11.7 W in argon, 11.2 W in helium, 11.0 W in oxygen, 11.3 W in air, and 12.1 W in unsparged water when the electrical input rose from 60 to 100 W. These values show that increasing the input power enlarged both the baseline thermal dissipation and the gas-dependent apparent cavitation increment. However, the relative cavitation fraction did not rise dramatically with power and even decreased in carbon dioxide because the total calorimetric signal increased while ΔP remained very small. This observation argues against the simplistic assumption that increasing electrical power necessarily increases the proportion of power converted into useful cavitation. Instead, the reactor retains a substantial non-cavitational channel even at higher input, and the extent to which the added power becomes chemically productive depends strongly on dissolved gas composition [3], [4].

Taken together, Fig. 2 establishes the energetic foundation of the entire manuscript. It shows that total power dissipation in the liquid depends strongly on electrical input, that dissolved gas exerts a real but moderate influence on this total, and that a large fraction of the calorimetric signal persists in the vacuum-treated gas-depleted reference and must therefore be assigned to non-cavitational dissipation. The ΔP framework then transforms this qualitative picture into a quantitative one by identifying a dominant non-cavitational baseline and a smaller, gas-dependent, apparent cavitation contribution. This is the most rigorous interpretation that calorimetry alone can support, and it provides the essential energetic reference needed to interpret why the later dosimetries and substrate degradation results diverge so strongly from the thermal ranking [2], [10].

The divergence between ΔP and the subsequent chemical readouts is therefore physically expected. ΔP is an energetic excess over the boiled/headspace-free/vacuum-treated reference and includes all gas-enabled pathways that end as heat in the liquid, including stable bubble oscillation, viscous damping, acoustic scattering, collapse heating, and bubble-cloud attenuation. The dosimetric probes, however, respond only to selected chemical consequences of bubble collapse. A high-γ, low-thermal-conductivity gas such as Ar can favor high-temperature collapse and short-lived interfacial radical attack, whereas O_2_ can redirect bubble chemistry toward oxygen-centered radicals, peroxide accumulation, and bulk oxidizing capacity. He can dissipate energy through thermally leaky bubbles because of its very high thermal conductivity, while CO_2_ promotes damped and weakly productive collapse. Consequently, similar ΔP values may lead to different KI, KI-AHM, Fricke, H_2_O_2_, 4-NP, SCL, and SSY responses because each readout samples a different spatial and chemical fraction of the cavitation field [3], [23].

### 3.2 KI and KI-AHM dosimetry

**Fig. 3:**
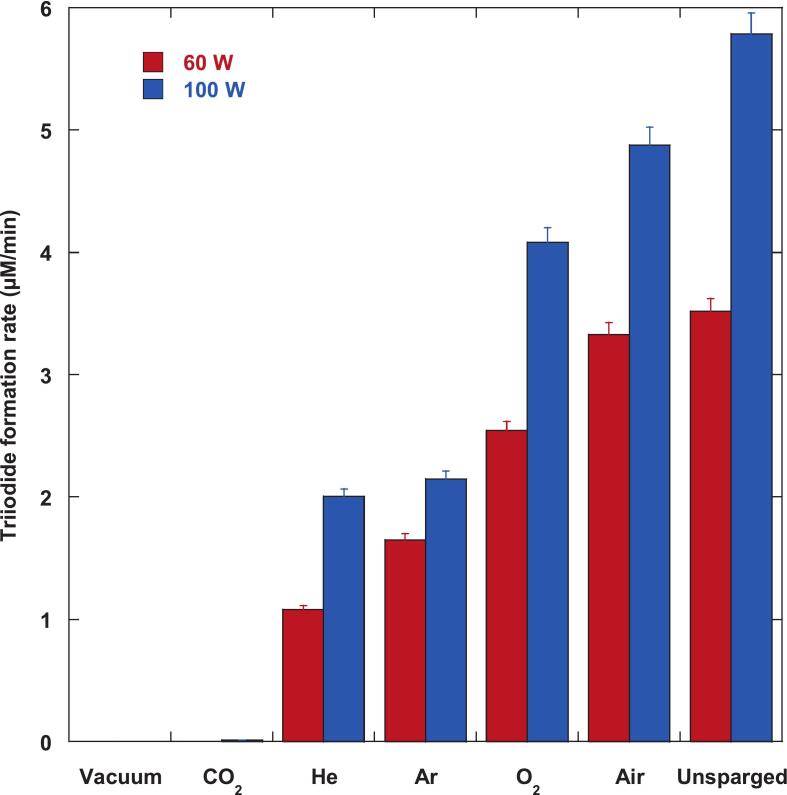
KI dosimetry in the vacuum-treated gas-depleted reference, unsparged, and gas-conditioned media at 60 and 100 W (volume: 200 mL, frequency: 425 kHz, temperature: 20 °C).

Fig. 3 shows that KI dosimetry is far more sensitive to dissolved gas composition than calorimetry. At 60 W, the triiodide formation rate was essentially zero in the vacuum-treated gas-depleted reference and CO_2_-conditioned water, then increased to about 1.08 μM/min in helium, 1.65 μM/min in argon, 2.54 μM/min in oxygen, 3.33 μM/min in air, and 3.51 μM/min in unsparged water. At 100 W, the same trend was amplified, with values of about 2.00 μM/min in helium, 2.15 μM/min in argon, 4.09 μM/min in oxygen, 4.88 μM/min in air, and 5.79 μM/min in unsparged water, while the vacuum-treated gas-depleted reference and CO_2_-conditioned water again remained nearly inactive. This ordering is highly significant because KI dosimetry does not report total dissipated power. It reports the oxidation of iodide to iodine followed by rapid conversion to triiodide, and is therefore a chemical readout of oxidizing species generated by acoustic cavitation, particularly near the bubble–liquid interface [6], [10]. The much broader separation among gases in Fig. 3, compared with the relatively compressed calorimetric ranking in Fig. 2, shows that dissolved gas composition affects the conversion of dissipated acoustic power into oxidizing chemistry much more strongly than it affects the total heat balance of the reactor [10], [20].

The contrast with calorimetry is the first major mechanistic point. Calorimetry showed that oxygen, helium, argon, air, and unsparged water occupied a relatively narrow range of about 19.2 to 20.8 W at 60 W and 30.4 to 32.9 W at 100 W, whereas KI dosimetry spread the same conditions over a much broader chemical interval. For example, at 100 W, helium and oxygen differed by only about 0.4 W in calorimetric power, yet their KI responses differed by roughly a factor of two, 2.00 versus 4.09 μM/min. Likewise, unsparged water exceeded air by only about 1.6 W calorimetrically, but its KI response was still appreciably higher, 5.79 versus 4.88 μM/min. These comparisons show that the gas-dependent calorimetric excess identified by the ΔP framework is not chemically uniform. The same apparent cavitation power increment can generate very different amounts of iodide oxidation depending on dissolved gas composition. In that sense, KI dosimetry reveals the chemical efficiency with which the gas-enabled fraction of calorimetric power is converted into interfacial oxidation [3], [10], [20].

This conclusion becomes even clearer when the KI data are normalized conceptually against the ΔP values derived from calorimetry. At 60 W, the apparent cavitation contribution was about 4.7 to 4.8 W in helium and oxygen, yet the KI response was approximately 1.08 μM/min in helium and 2.54 μM/min in oxygen. At 100 W, the corresponding ΔP values were about 6.4 W in helium and 6.3 W in oxygen, while the KI rates were about 2.00 and 4.09 μM/min, respectively. In practical terms, oxygen produced nearly twice as much KI response as helium for essentially the same apparent cavitation power increment. Air and unsparged water were even more effective. This demonstrates that the apparent cavitation contribution resolved by ΔP analysis must not be interpreted as a single universal cavitation number. It is an energetic quantity whose chemical expression depends strongly on the gas environment and on the oxidizing pathways available in the liquid [10], [20].

This behavior can be linked directly to the spatial selectivity of iodide oxidation. KI responds primarily to oxidizing species that survive long enough to reach the bubble–liquid interface and react with iodide. Oxygen-containing media therefore give disproportionately high KI responses because O_2_ participates in the bubble and interfacial radical network, favoring oxygen-centered intermediates and oxidizing pathways that are efficiently expressed at the interface. Helium, although calorimetrically comparable to oxygen in some cases, is less effective chemically because its high thermal conductivity promotes heat leakage from the bubble interior, reducing hot-spot severity and limiting radical productivity. Argon favors more severe collapse than helium, but in KI alone it does not benefit from the same oxygen-mediated interfacial oxidation chemistry. Thus, KI demonstrates that ΔP becomes chemically productive only when bubble interior physics and interfacial oxidant transfer are favorable [3], [23]. Because dissolved-gas concentrations were not measured after irradiation, the KI response is not interpreted as a function of residual gas concentration. It is instead interpreted as the chemical outcome of the initial gas-conditioning state and the subsequent cavitation dynamics generated under identical ultrasonic operating conditions.

The very low response under carbon dioxide deserves particular attention. Carbon dioxide was only slightly above the vacuum-treated reference in calorimetry, with ΔP values of about 1.8 W at 60 W and 1.3 W at 100 W, and its KI response was essentially negligible at both powers. This is consistent with the known role of carbon dioxide in moderating cavitation. Dissolved gas properties determine bubble composition, damping, thermal conductivity, and collapse chemistry, and carbon dioxide is well known to suppress the most intense cavitation events and the associated radical chemistry [20]. The present KI data show that this suppressive effect extends beyond chemistry alone and is already visible at the level of the apparent cavitation power increment defined by ΔP. In other words, carbon dioxide does not simply reduce iodide oxidation. It constrains the fraction of total calorimetric power that becomes chemically productive cavitation [20].

**Fig. 4:**
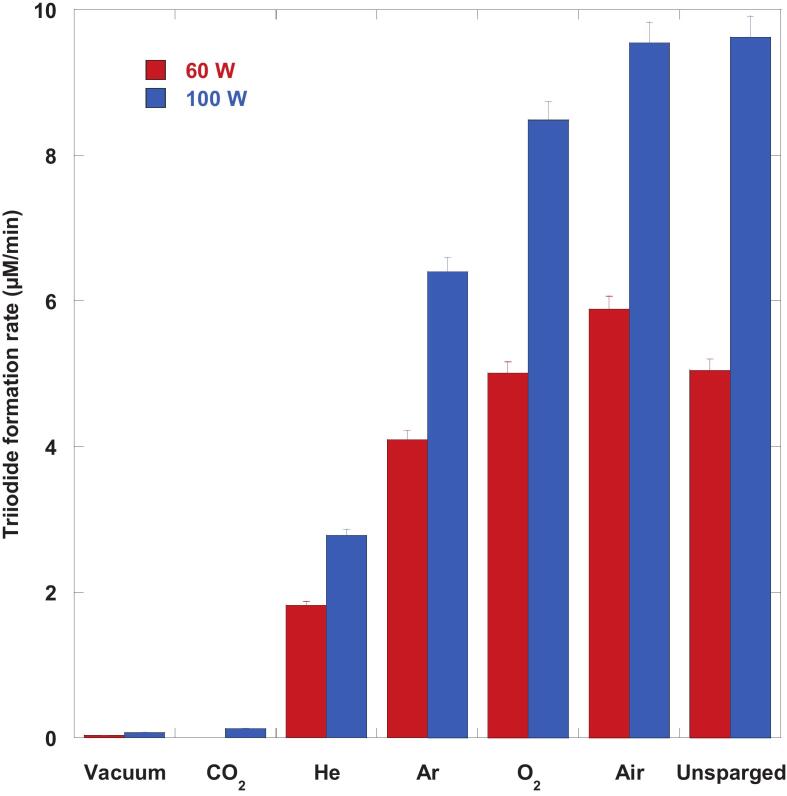
KI-AHM dosimetry in the vacuum-treated gas-depleted reference, unsparged, and gas-conditioned media at 60 and 100 W (volume: 200 mL, frequency: 425 kHz, temperature: 20 °C).

Fig. 4 shows that the KI-AHM assay amplifies the gas effect even further. At 60 W, the triiodide formation rate was again negligible in the vacuum-treated gas-depleted reference and CO_2_-conditioned water, then increased to about 1.82 μM/min in helium, 4.08 μM/min in argon, 5.02 μM/min in oxygen, 5.90 μM/min in air, and 5.03 μM/min in unsparged water. At 100 W, the response increased to about 2.78 μM/min in helium, 6.40 μM/min in argon, 8.49 μM/min in oxygen, 9.53 μM/min in air, and 9.62 μM/min in unsparged water. The significance of this assay is that KI-AHM does not monitor iodide oxidation alone. In the recent coupled calorimetric and multidosimetric framework at 425 kHz, KI was interpreted as a probe of interfacial hydroxyl radical chemistry, whereas KI-AHM was shown to capture hydroxyl radicals together with the hydrogen peroxide branch of the oxidation chemistry [10]. Fig. 4 therefore indicates that a substantial fraction of the oxidizing inventory generated during ultrasonication is expressed not only as direct iodide oxidation, but also as peroxide-supported oxidation that becomes visible only in the catalyzed system [10].

The KI-AHM response provides a second probe-specific link between bubble physics and chemistry. The AHM-catalyzed assay amplifies the contribution of H_2_O_2_-derived oxidation, and therefore becomes more sensitive to gases that favor radical recombination and peroxide accumulation. Oxygen and air promote this branch because oxygenated bubble interiors and interfacial regions facilitate the conversion of primary radicals into longer-lived oxidants. Argon can also generate a strong KI-AHM response because intense collapse produces high radical densities that subsequently recombine to H_2_O_2_. In contrast, helium remains weak because thermal leakage limits radical formation, while CO_2_ remains nearly inactive because damping and collapse cushioning suppress radical-productive cavitation. The difference between KI and KI-AHM therefore reflects not only a change in analytical chemistry, but also a change in the bubble-derived pathway being sampled [3], [23].

The difference between Fig. 3, Fig. 4 is quantitatively revealing. At 60 W, the KI-AHM response exceeded the KI response by about 0.74 μM/min in helium, 2.43 μM/min in argon, 2.48 μM/min in oxygen, 2.57 μM/min in air, and 1.52 μM/min in unsparged water. At 100 W, these increments increased to approximately 0.78, 4.25, 4.40, 4.65, and 3.83 μM/min, respectively. Expressed as KI-AHM to KI ratios, the catalytic amplification at 100 W was about 1.39 in helium, 2.98 in argon, 2.08 in oxygen, 1.95 in air, and 1.66 in unsparged water. These values show that the peroxide-related branch was weak in helium, extremely pronounced in argon, and strongly developed in oxygen, air, and unsparged water. This is a key mechanistic result. The chemical gain introduced by AHM is not uniform across gases, which means that the gas-dependent calorimetric increment is partitioned differently among direct interfacial oxidation and peroxide-supported oxidation depending on dissolved gas composition [6], [10].

The comparison with calorimetry reinforces this point. At 100 W, oxygen, helium, and argon had very similar calorimetric powers, all close to 30.4 to 30.9 W, yet the KI-AHM responses were approximately 8.49, 2.78, and 6.40 μM/min, respectively. Thus, the thermal ranking alone cannot explain the chemical ranking. When these rates are considered relative to the ΔP values, the contrast becomes even sharper. Oxygen produced about 1.35 μM/min·W of apparent cavitation power, air about 1.32 μM/min·W, unsparged water about 1.09 μM/min·W, argon about 0.94 μM/min·W, and helium only about 0.43 μM/min·W. At 60 W the same trend was already visible, with oxygen and air both near or above 1.0 μM/min·W of ΔP, argon near 0.89 μM/min·W, unsparged water near 0.81 μM/min·W, and helium only about 0.39 μM/min·W. These values show that oxygen-containing and naturally equilibrated media use the apparent cavitation power increment much more efficiently for combined hydroxyl radical and peroxide-supported oxidation than helium does, while argon occupies an intermediate position [10], [20].

The gas ranking itself is also mechanistically informative. In KI alone, the strongest condition was unsparged water, followed closely by air and oxygen. In KI-AHM, air became the highest condition at 60 W, while at 100 W air and unsparged water were nearly identical and oxygen remained only slightly lower. This suggests that oxygen-containing and naturally equilibrated media favor a broader oxidizing environment than KI alone can reveal. By contrast, helium remained consistently low in both KI and KI-AHM, despite having a calorimetric power comparable to oxygen, argon, and air. The implication is that helium supports total energy dissipation without converting that power efficiently into either direct iodide oxidation or peroxide-supported oxidation. Such behavior is fully consistent with the literature showing that dissolved gas composition strongly affects cavitation chemistry through its influence on bubble thermodynamics and gas-phase chemistry, and that gas effects on chemical oxidation can be much stronger than gas effects on calorimetry [3], [20].

Taken together, Fig. 3, Fig. 4 provide the first direct chemical validation of the ΔP-based energetic partitioning established from calorimetry. Fig. 2 showed that a substantial non-cavitational baseline persists in the vacuum-treated gas-depleted reference and that the gas-dependent apparent cavitation contribution remains a minority fraction of the total heat balance. The KI assays now show that this apparent cavitation contribution is chemically heterogeneous. In oxygen-containing and unsparged media, a given ΔP value yields a relatively large interfacial oxidation response and a strong peroxide-supported amplification. In helium, the same energetic framework yields only modest chemical activity. In carbon dioxide, both the energetic increment and the chemical response are nearly extinguished. The conclusion is therefore not simply that dissolved gas changes cavitation intensity. Rather, dissolved gas governs how the apparent cavitation-dependent fraction of calorimetric power is distributed between direct iodide oxidation and peroxide-assisted oxidation, and that distribution cannot be inferred from calorimetry alone [6], [10], [20].

Fig. 3, Fig. 4 demonstrate that iodide-based dosimetry resolves the chemical meaning of the ΔP defined apparent cavitation contribution, showing that oxygen-containing and unsparged media convert gas-enabled calorimetric power into interfacial oxidation and peroxide-supported oxidizing capacity far more efficiently than would be inferred from calorimetry alone [10], [20].

### 3.3 Fricke and H_2_O_2_ dosimetry

**Fig. 5:**
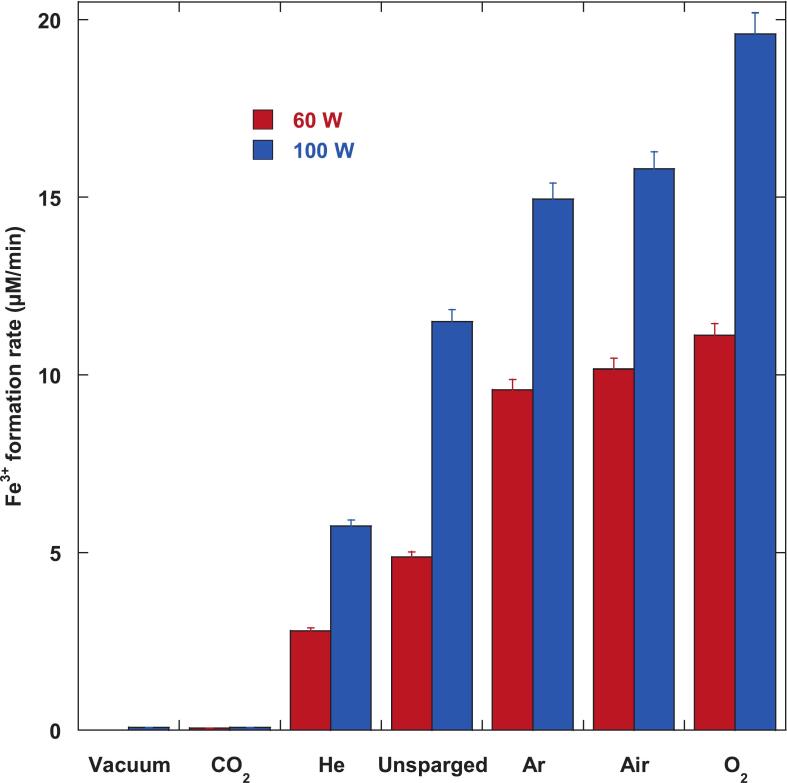
Fricke dosimetry in the vacuum-treated gas-depleted reference, unsparged, and gas-conditioned media at 60 and 100 W (volume: 200 mL, frequency: 425 kHz, temperature: 20 °C).

Fig. 5 shows that Fricke dosimetry responds to dissolved gas composition much more sharply than calorimetry and in a different order than KI and KI-AHM. At 60 W, the Fe^3+^ formation rate was essentially zero in the vacuum-treated gas-depleted reference and CO_2_-conditioned water, then increased to about 2.8 μM/min in helium, 4.9 μM/min in unsparged water, 9.6 μM/min in argon, 10.2 μM/min in air, and 11.1 μM/min in oxygen. At 100 W, the same order was preserved but the separation became larger, with values of about 5.8 μM/min in helium, 11.6 μM/min in unsparged water, 14.9 μM/min in argon, 15.9 μM/min in air, and 19.6 μM/min in oxygen, while the vacuum-treated gas-depleted reference and CO_2_-conditioned water again remained negligible. This pattern demonstrates that Fricke dosimetry is not governed by total power dissipation alone. Rather, it resolves the fraction of acoustic energy that is expressed as oxidizing species capable of converting Fe^2+^ into Fe^3+^ in the liquid phase [6], [10], [21]. The much larger spread of the Fricke data, compared with the relatively narrow calorimetric grouping of the active gas conditions, therefore shows that gas composition has a far stronger effect on oxidative chemistry than on the total heat balance of the reactor [3], [10].

The comparison with calorimetry is immediately revealing. In the calorimetric results, oxygen, helium, argon, and air occupied a narrow range of about 19.2 to 20.0 W at 60 W and 30.4 to 31.3 W at 100 W, while unsparged water gave the highest total power in liquid at about 20.8 and 32.9 W. Fig. 5 does not follow that order. Oxygen becomes the strongest condition, argon and air follow, and unsparged water falls into an intermediate position. Thus, the condition that maximizes total dissipated power does not maximize oxidative conversion of Fe^2+^. Within the ΔP-based energetic partitioning framework, this means that oxygen converts the apparent cavitation contribution into oxidizing chemistry more efficiently than unsparged water, even though unsparged water gives the largest total calorimetric signal [10], [15]. This decoupling is one of the most important outcomes of the combined analysis, because it shows that energetic performance and chemical performance are related but not identical descriptors of reactor behavior [3], [10], [15].

The contrast with KI and KI-AHM is equally instructive. KI dosimetry favored unsparged water, then air and oxygen, whereas KI-AHM further amplified oxygen, air, and unsparged media by incorporating the peroxide-assisted oxidation pathway. Fricke dosimetry shifts the maximum clearly toward oxygen. This shift carries a strong mechanistic implication. KI primarily reflects oxidation available to iodide at or near the bubble–liquid interface, while KI-AHM extends that information by capturing hydrogen peroxide-assisted iodide oxidation. Fricke, by contrast, reports a broader oxidizing environment in the liquid. The stronger dominance of oxygen in Fig. 5 therefore indicates that oxygen-rich conditions do not simply increase interfacial oxidation. They favor the formation and expression of oxidizing equivalents in the bulk liquid to a greater extent than argon, helium, or unsparged water [6], [10], [21]. Comparative dosimetry studies have reported the same general point, namely that KI, Fricke, and hydrogen peroxide dosimetries do not provide interchangeable rankings because they interrogate distinct branches of sonochemical oxidation [6], [21].

A numerical comparison against the ΔP values derived from calorimetry clarifies this point further. At 60 W, the apparent cavitation contribution was about 4.8 W in oxygen, 4.6 W in argon, 5.4 W in air, and 6.2 W in unsparged water, yet the corresponding Fricke rates were about 11.1, 9.6, 10.2, and 4.9 μM/min, respectively. At 100 W, the apparent cavitation contribution was about 6.3 W in oxygen, 6.8 W in argon, 7.2 W in air, and 8.8 W in unsparged water, while the Fricke rates were about 19.6, 14.9, 15.9, and 11.6 μM/min, respectively. Expressed as apparent Fricke productivity per watt of ΔP, oxygen gave approximately 2.31 μM/min·W at 60 W and 3.11 μM/min·W at 100 W. The corresponding values were about 2.08 and 2.21 for air, 2.09 and 2.19 for argon, and only 0.91 and 1.32 for unsparged water. These numbers show that the apparent cavitation power increment identified from calorimetry is chemically far more productive under oxygen-rich conditions than under unsparged conditions when the oxidative branch is evaluated by Fricke dosimetry [6], [10].

The strong position of oxygen relative to argon deserves particular emphasis. Argon is often associated with more violent bubble collapse because of its monoatomic character and low thermal conductivity, and this expectation is indeed reflected later by the 4-NP results. Fricke dosimetry, however, places oxygen above argon. This indicates that the Fe^3+^ response is not controlled only by the hottest collapse events. It also depends on the broader oxidizing chemistry generated in oxygen-containing media. Comparative high-frequency dosimetry studies have noted that Fricke can capture not only hydroxyl radical related oxidation but also contributions from oxygen-centered species, especially in oxygen-rich systems [21]. The present ranking is therefore entirely plausible. Oxygen does not necessarily maximize only the physical violence of collapse. It maximizes the oxidizing environment expressed in the Fricke reagent [3], [21].

**Fig. 6:**
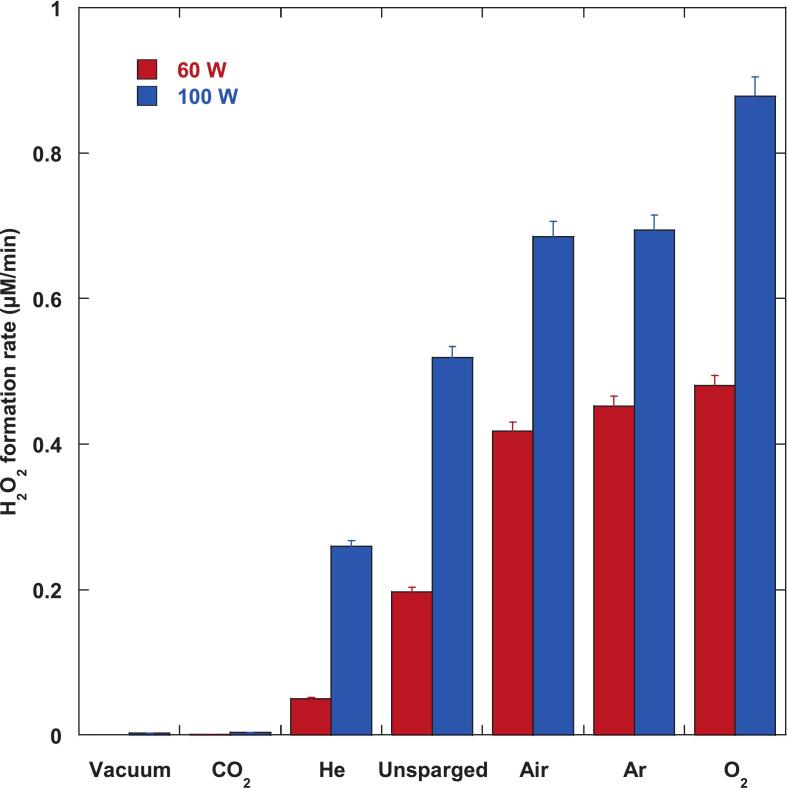
Hydrogen peroxide dosimetry in the vacuum-treated gas-depleted reference, unsparged, and gas-conditioned media at 60 and 100 W (volume: 200 mL, frequency: 425 kHz, temperature: 20 °C).

Fig. 6 shows that hydrogen peroxide dosimetry follows a similar but not identical pattern. At 60 W, H_2_O_2_ formation was essentially zero in the vacuum-treated gas-depleted reference and CO_2_-conditioned water, then reached about 0.06 μM/min in helium, 0.19 μM/min in unsparged water, 0.42 μM/min in air, 0.45 μM/min in argon, and 0.48 μM/min in oxygen. At 100 W, the same ranking was retained, with about 0.26 μM/min in helium, 0.50 μM/min in unsparged water, 0.69 μM/min in air, 0.70 μM/min in argon, and 0.88 μM/min in oxygen, while the vacuum-treated gas-depleted reference and CO_2_-conditioned water again remained almost inactive. Hydrogen peroxide is not a primary radical but an accumulated molecular product of radical recombination. Fig. 6 therefore highlights the recombination branch of sonochemical oxidation and confirms that this branch is also strongly favored by oxygen-rich conditions [6], [10], [15].

The combined Fricke/H_2_O_2_ behavior provides a clear example of why ΔP cannot be mapped directly onto a single chemical yield. Fricke dosimetry reports a broad oxidizing inventory capable of converting Fe^2+^ to Fe^3+^, whereas H_2_O_2_ dosimetry reports the accumulated product of radical recombination. Oxygen-rich bubbles favor both responses because O_2_ participates in the hot bubble and interfacial reaction network, producing oxygen-centered radical pathways and stabilizing oxidizing equivalents that can persist into the bulk liquid. Argon can generate severe collapse and high instantaneous radical densities, but it does not provide the same oxygen-rich chemistry that maximizes bulk Fe^2+^ oxidation and peroxide accumulation. Helium is less effective because thermal leakage reduces the radical inventory, and CO_2_ remains suppressed because damped collapse produces little chemically useful oxidizing flux. Thus, Fricke and H_2_O_2_ convert the ΔP-defined energetic contribution into bulk oxidizing capacity and recombination chemistry, rather than into a direct measure of collapse intensity [3], [23]. As for the KI assays, these trends are not presented as concentration-normalized gas effects because the dissolved concentrations of the gases before and after irradiation were not analytically determined. The comparison is therefore based on standardized gas conditioning and identical ultrasonication conditions.

The relation between Fig. 5, Fig. 6 is mechanistically very useful. Both dosimetries rank oxygen first and both suppress the vacuum-treated gas-depleted reference and CO_2_-conditioned water, confirming that the vacuum calorimetric baseline corresponds largely to non-cavitational dissipation from a chemical standpoint. However, the oxygen preference is even sharper in Fricke than in hydrogen peroxide. At 100 W, oxygen exceeded argon by about 4.7 μM/min in Fricke but by only about 0.18 μM/min in hydrogen peroxide. This difference implies that Fricke captures a broader oxidizing inventory than H_2_O_2_ alone. Rajamma et al. [21] pointed out that Fricke can include contributions from oxygen-centered radicals in addition to pathways closely related to hydroxyl radical chemistry. Hydrogen peroxide dosimetry, by contrast, isolates one accumulated product and therefore gives a more selective view of the recombination branch [6], [15], [21]. In the present data, O_2_-conditioned water appears to maximize both the broad oxidizing environment and the specific peroxide branch, but the extent of enhancement is greater for the former than for the latter [6], [15], [21].

The link with KI-AHM is especially strong. In the KI-AHM assay, the catalytic increment over KI alone was largest in oxygen, air, argon, and unsparged water, which suggested a major contribution from peroxide-assisted oxidation. Fig. 6 now provides direct support for that interpretation. At 100 W, oxygen, air, argon, and unsparged water showed H_2_O_2_ rates of about 0.88, 0.69, 0.70, and 0.50 μM/min, respectively, while helium remained much lower at about 0.26 μM/min. The same gases that showed the largest KI-AHM amplification are therefore the gases that also produced the largest hydrogen peroxide accumulation. This agreement confirms that the difference between KI and KI-AHM genuinely reflects the peroxide branch of the chemistry rather than only a change in iodide response [10], [15]. It also shows that oxygen-rich conditions do not merely support direct oxidation. They favor both direct oxidizing chemistry and the accumulation of more stable oxidizing products in the liquid [10], [15].

When hydrogen peroxide formation is interpreted relative to ΔP, the same energetic conclusion emerges as in the Fricke analysis, although the absolute values are naturally much smaller. At 100 W, oxygen produced about 0.14 μM/min·W of H_2_O_2_ per watt of apparent cavitation power, compared with about 0.10 for argon, 0.10 for air, 0.06 for unsparged water, and 0.04 for helium. At 60 W, oxygen gave about 0.10 μM/min·W, argon and air about 0.08, unsparged water about 0.03, and helium only about 0.01. Thus, even when the calorimetric excess is similar, oxygen converts that excess into peroxide formation more efficiently than the other gases. The implication is that the gas-dependent apparent cavitation contribution resolved by calorimetry is chemically redistributed in favor of peroxide accumulation under oxygen-rich conditions [10], [15].

Taken together, Fig. 5, Fig. 6 show that Fricke and hydrogen peroxide dosimetries shift the interpretation from interfacial oxidation, emphasized by KI, toward bulk oxidizing capacity and recombination chemistry. The vacuum-treated gas-depleted reference and CO_2_-conditioned water remain almost inactive despite a measurable calorimetric signal, confirming that these two assays are genuinely cavitation selective. Helium supports some chemical activity, but far less than oxygen, air, or argon. Oxygen-conditioned water gives the highest response in both dosimetries, which shows that oxygen does not simply accompany cavitation but actively reshapes the chemistry of the collapse products. Within the ΔP-based energetic partitioning framework, oxygen is therefore the gas that most effectively converts apparent cavitation power into bulk oxidizing capacity and peroxide accumulation, whereas unsparged water remains the condition that maximizes total dissipated power and KI-based oxidation [3], [6], [10], [15], [21]. This divergence among rankings is not contradictory. It is the clearest evidence that the same apparent cavitation power increment can be partitioned among distinct chemical pathways depending on dissolved gas composition [3], [10].

Fig. 5, Fig. 6 demonstrate that Fricke and hydrogen peroxide dosimetries resolve oxidative branches that are only partially visible in KI-based assays and cannot be predicted from calorimetry alone, with O_2_-conditioned water emerging as the most efficient condition for converting the ΔP defined apparent cavitation contribution into bulk oxidizing capacity and peroxide accumulation [3], [6], [10], [15], [21].

### 3.4 4-nitrophenol dosimetry

**Fig. 7:**
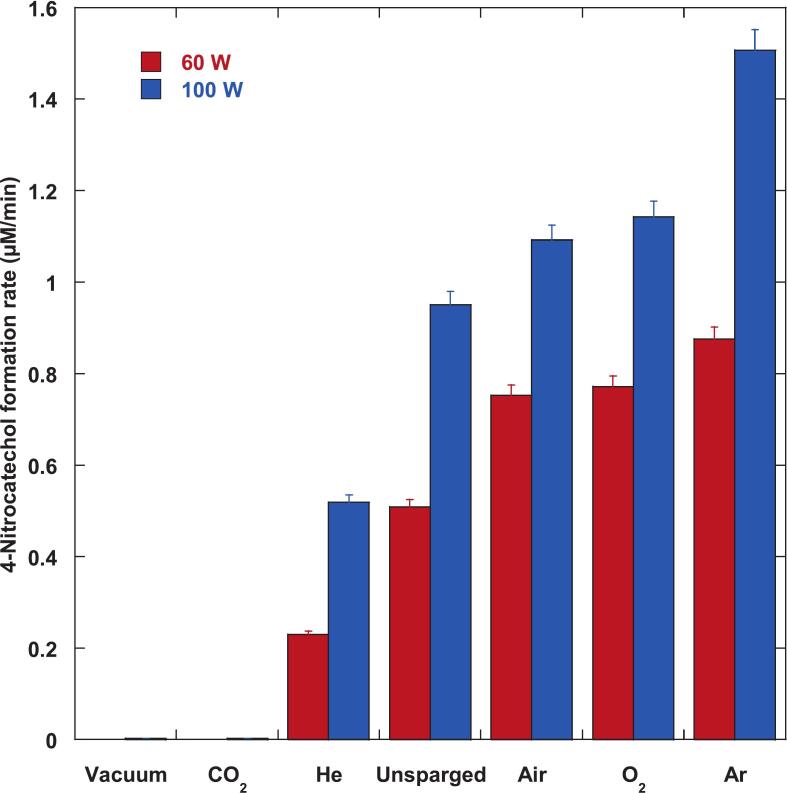
4-Nitrophenol dosimetry in the vacuum-treated gas-depleted reference, unsparged, and gas-conditioned media at 60 and 100 W (volume: 200 mL, frequency: 425 kHz, temperature: 20 °C).

Fig. 7 shows that 4-NP dosimetry, expressed here as the formation rate of 4-NC, yields a gas ranking that is clearly different from the rankings obtained by calorimetry, KI, KI-AHM, Fricke, and hydrogen peroxide dosimetries. At 60 W, the 4-NC formation rate was essentially negligible in the vacuum-treated gas-depleted reference and CO_2_-conditioned water, then increased to about 0.23 μM/min in helium, 0.51 μM/min in unsparged water, 0.75 μM/min in air, 0.77 μM/min in oxygen, and 0.88 μM/min in argon. At 100 W, the same trend was amplified, with values of about 0.52 μM/min in helium, 0.95 μM/min in unsparged water, 1.09 μM/min in air, 1.14 μM/min in oxygen, and 1.51 μM/min in argon, while the vacuum-treated gas-depleted reference and CO_2_-conditioned water again remained nearly inactive. This ordering is highly significant because hydroxylation of 4-NP to 4-NC is widely used as a selective probe of near-interface hydroxyl radical attack rather than a measure of total dissipated acoustic power [10], [11]. Recent calorimetry-anchored multidosimetric work at 425 kHz explicitly interpreted 4-NP hydroxylation as a selective near interface hydroxyl radical probe that triangulates where delivered energy is directed chemically [10].

The preferential response of 4-NP under argon can be understood from the specific bubble physics required for direct aromatic hydroxylation. Formation of 4-NC requires highly reactive short-lived hydroxylating species near the bubble–liquid interface, and this pathway is favored by intense collapse events rather than by total heat generation alone. Argon is well suited to this response because its high heat capacity ratio and low thermal conductivity favor stronger compression heating and less thermal leakage during collapse. Oxygen and air also generate oxidative chemistry, but part of their chemistry is redirected toward oxygen-centered radicals and peroxide-forming pathways, which are more strongly reflected in Fricke, H_2_O_2_, and KI-AHM assays than in direct aromatic hydroxylation. Helium dissipates energy through thermally leaky collapse, while CO_2_ suppresses violent collapse. Therefore, the 4-NP ranking demonstrates that the most chemically aggressive interfacial hydroxylation events can be maximized under a gas condition that is not the calorimetric maximum [3], [23].

The comparison with calorimetry is the first key mechanistic point. Fig. 2 showed that unsparged water produced the highest total power in liquid, with calorimetric values of about 20.8 W at 60 W and 32.9 W at 100 W, whereas oxygen, helium, argon, and air formed a narrow upper group. Fig. 7 does not reproduce that order. Instead, argon becomes the leading condition in 4-NP dosimetry, oxygen and air follow, and unsparged water falls into an intermediate position. This divergence demonstrates that the condition maximizing total dissipated power is not the condition maximizing aromatic hydroxylation chemistry. Within the ΔP-based energetic partitioning framework, the signal measured in the vacuum-treated gas-depleted reference defines a lower bound for the non-cavitational baseline, while the gas-dependent excess defines the apparent cavitation contribution. Fig. 7 then shows that this apparent cavitation contribution is chemically heterogeneous: similar ΔP values can produce very different 4-NC yields depending on dissolved gas composition. In practical terms, calorimetry quantifies how much power is dissipated in the liquid, whereas 4-NP dosimetry reveals how efficiently that gas-enabled fraction is converted into direct aromatic hydroxylation [3], [10].

A quantitative comparison against the calorimetric ΔP values makes this distinction explicit. At 60 W, the apparent cavitation contribution was about 4.6 W in argon, 4.8 W in oxygen, 5.4 W in air, and 6.2 W in unsparged water, yet the corresponding 4-NC formation rates were about 0.88, 0.77, 0.75, and 0.51 μM/min, respectively. At 100 W, the ΔP values were about 6.8 W in argon, 6.3 W in oxygen, 7.2 W in air, and 8.8 W in unsparged water, while the corresponding rates were about 1.51, 1.14, 1.09, and 0.95 μM/min, respectively. Expressed as apparent 4-NP productivity per watt of ΔP, argon gave approximately 0.19 μM/min·W at 60 W and 0.22 μM/min·W at 100 W, compared with about 0.16 and 0.18 for oxygen, 0.14 and 0.15 for air, and only 0.08 and 0.11 for unsparged water. These values show that argon converts the apparent cavitation contribution into direct aromatic hydroxylation more efficiently than any other gas condition, even though it does not maximize the total calorimetric signal [3], [10].

The relation to KI and KI-AHM dosimetries is especially instructive. KI favored unsparged water, then air and oxygen, while KI-AHM further amplified oxygen-containing and unsparged media by incorporating the peroxide-assisted oxidation branch. Fig. 7 shifts the maximum toward argon. This shift is mechanistically meaningful because the iodide assays and the 4-NP assay interrogate different stages of the energy-to-chemistry cascade. KI is generally interpreted as a probe of interfacial iodide oxidation by hydroxyl radical chemistry, while KI-AHM extends that response by incorporating hydrogen peroxide-assisted oxidation. By contrast, 4-NP dosimetry tracks hydroxylation of an aromatic substrate into 4-NC. The higher relative position of argon in Fig. 7 therefore suggests that argon favors the subset of chemically aggressive collapses that promote direct hydroxyl radical attack on the aromatic probe, whereas oxygen-containing and unsparged media favor a larger integrated oxidant inventory that is more effectively captured by iodide chemistry [6], [10]. This difference is entirely consistent with recent multidosimetric analyses showing that KI, KI-AHM, hydrogen peroxide, and 4-NP report distinct but complementary branches of cavitation chemistry rather than one single universal oxidative response [6], [10].

The comparison with Fricke and hydrogen peroxide dosimetries strengthens this interpretation. Fricke and hydrogen peroxide both placed oxygen at the top, followed by air and argon, which indicated that O_2_-conditioned water maximizes bulk oxidizing capacity and the recombination branch that forms hydrogen peroxide. Fig. 7 does not follow that ordering. Argon overtakes oxygen and air, while unsparged water again remains intermediate. This means that 4-NP dosimetry is less influenced by the total oxidant inventory stabilized in the liquid and more influenced by the subset of cavitation events that generate hydroxyl radicals able to hydroxylate the aromatic substrate before they are diverted into recombination or oxygen-centered product channels. In other words, Fricke and hydrogen peroxide emphasize bulk oxidizing expression, whereas 4-NP emphasizes direct aromatic hydroxylation by short-lived radical attack near cavitating interfaces [6], [10]. The difference among these rankings is exactly what a multidosimetric framework is expected to reveal, and recent comparative work has indeed found that 4-NP can behave differently from KI, Fricke, and hydrogen peroxide, particularly when operating conditions change [6], [11].

The dominance of argon in Fig. 7 is fully consistent with the established effect of dissolved gas on cavitation bubble thermodynamics. Dissolved gas type alters the adiabatic ratio, thermal conductivity, mass transfer, and chemical participation inside the collapsing bubble, and these changes strongly influence hot spot conditions and radical escape into the surrounding liquid. Noble gases are well known to favor more severe collapse because of their thermophysical properties, whereas gases such as carbon dioxide damp or chemically moderate the most intense cavitation conditions. The fact that argon is only one member of the upper calorimetric group but becomes the leading condition in 4-NP dosimetry therefore suggests that 4-NC formation is especially sensitive to the most chemically aggressive collapse events rather than to total dissipated power alone [3]. This interpretation is also consistent with earlier sonolysis work on aqueous 4-NP in argon-conditioned solution, which explicitly linked the reaction to hydroxyl radicals formed in cavitation bubbles and escaping into the aqueous phase [22].

The weak response of helium provides an equally useful contrast. Helium remained clearly above the vacuum-treated gas-depleted reference and CO_2_-conditioned water, which confirms that it supported cavitation, but its 4-NC formation rate was much lower than that of argon and also below those of oxygen and air. This result indicates that the mere presence of cavitation is not sufficient to guarantee strong aromatic hydroxylation. The radical attack measured by 4-NP dosimetry depends on how the gas environment shapes collapse severity and radical availability at the bubble–liquid boundary. Helium therefore behaves as a condition that dissipates a comparable total acoustic power to the other active gases, but converts that power inefficiently into the direct aromatic hydroxylation pathway detected by the 4-NP assay [3], [10]. Such a decoupling is one of the clearest demonstrations that calorimetry and dosimetry cannot be interpreted interchangeably [3], [6], [10].

Carbon dioxide and vacuum define the other end of the spectrum. Both conditions are almost inactive in Fig. 7, despite the fact that calorimetry showed a substantial thermal signal in the vacuum-treated gas-depleted reference and a slight increase above the vacuum-treated reference in CO_2_-conditioned water. This contrast confirms that 4-NP dosimetry is genuinely cavitation selective. The thermal signal in the vacuum-treated gas-depleted reference belongs largely to non-cavitational dissipation, while the negligible 4-NC signal indicates that very little of that dissipation is expressed as aromatic hydroxylation chemistry. Carbon dioxide behaves similarly, showing that even when a small gas-dependent calorimetric increment exists, it may remain chemically unproductive if the gas suppresses the formation of highly reactive cavitation events. This result is fully aligned with the broader literature, which identifies carbon dioxide as a gas that often reduces radical production by altering bubble composition and collapse dynamics [3].

The effect of increasing electrical power from 60 to 100 W provides an additional mechanistic insight. All active gas conditions showed higher 4-NC formation at 100 W, but the relative increase was strongest for argon, which rose from about 0.88 to 1.51 μM/min, corresponding to an increase of about 72%. Oxygen increased from about 0.77 to 1.14 μM/min, about 48%, air from about 0.75 to 1.09 μM/min, about 45%, unsparged water from about 0.51 to 0.95 μM/min, about 87%, and helium from about 0.23 to 0.52 μM/min, about 126%, although from a much lower baseline. These increases are larger than would be inferred from calorimetry alone. This means that raising the electrical input does not merely increase the total heat balance, but selectively amplifies the fraction of cavitation that is capable of driving direct aromatic hydroxylation. Recent reactor characterization studies using 4-NP oxidation as a performance probe have likewise shown that local cavitation intensification can increase 4-NC yields much more strongly than calorimetric power alone [25].

Taken together, Fig. 7 provides the strongest evidence so far that the apparent cavitation contribution identified by ΔP analysis cannot be treated as chemically uniform. Unsparged water maximizes total dissipated power. KI and KI-AHM emphasize interfacial oxidation and peroxide-assisted oxidation, with strong responses in unsparged and oxygen-containing media. Fricke and hydrogen peroxide favor oxygen because oxygen promotes bulk oxidizing capacity and peroxide accumulation. Fig. 7, however, shifts the maximum toward argon, which shows that direct aromatic hydroxylation by hydroxyl radicals is favored under the gas environment that most strongly enhances chemically aggressive collapse events. The 4-NP assay therefore occupies a crucial position in the overall framework. It bridges reactor energetics and substrate-level radical attack by showing that the route from acoustic power to chemical reactivity is pathway-specific and profoundly controlled by dissolved gas composition [3], [6], [10].

Fig. 7 demonstrates that 4-NP dosimetry resolves a direct aromatic hydroxylation pathway that is only partially reflected by KI, KI-AHM, Fricke, or hydrogen peroxide measurements and is not predictable from calorimetry alone, with argon emerging as the most efficient gas for converting the apparent cavitation contribution into 4-NC formation [3], [10], [22].

### 3.5 Sonochemiluminescence

**Fig. 8:**
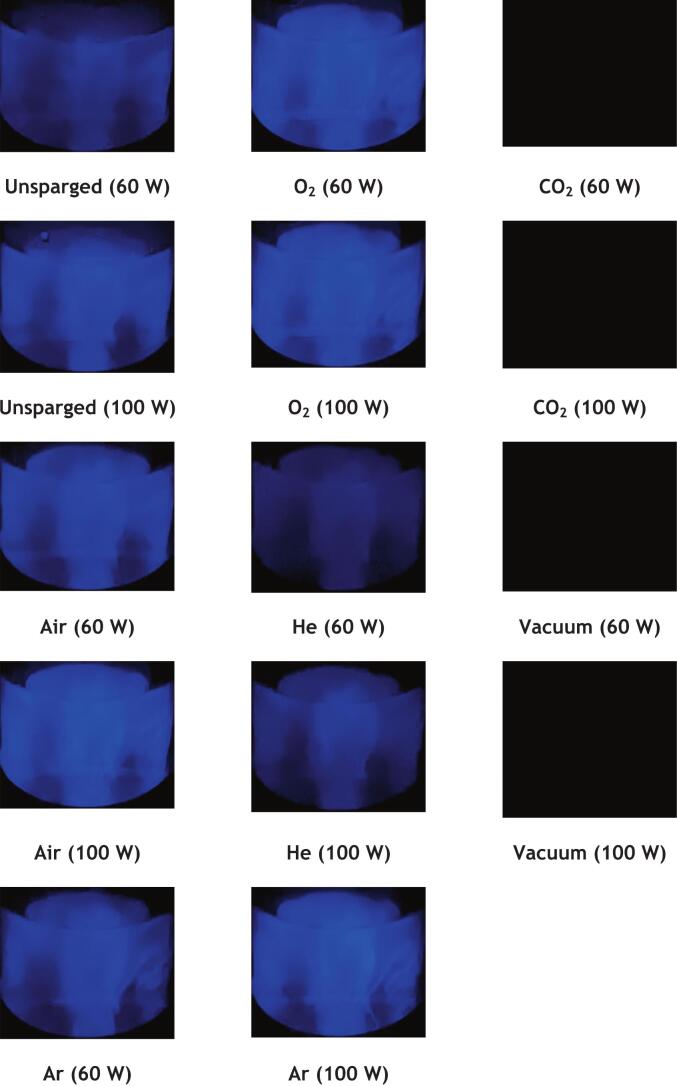
Representative SCL images obtained under different gas-conditioning states at 60 and 100 W (volume: 200 mL, frequency: 425 kHz, temperature: 20 °C).

Fig. 8 shows that SCL provides a direct visual confirmation of the gas-dependent cavitation field inferred from calorimetry and chemical dosimetry. The images reveal intense blue emission in unsparged water, O_2_-conditioned water, air-conditioned water, and Ar-conditioned water, weaker but still clearly visible emission in He-conditioned water, and almost no detectable emission in CO_2_-conditioned water and in the vacuum-treated gas-depleted reference at both 60 and 100 W. The overall brightness also increases when the electrical input is raised from 60 to 100 W, especially for oxygen, air, argon, and unsparged water. This behavior is fully consistent with the accepted interpretation of SCL as a diagnostic of active cavitation and local oxidizing conditions in luminol-containing media [3], [10], [27]. Since luminol light emission depends on reactive species generated during cavitation, especially under alkaline conditions, these images do not simply show where power is dissipated in the reactor. They show where the cavitation field becomes chemically emissive [3], [10]. The SCL images are therefore interpreted as qualitative spatial evidence of chemically active cavitation under each standardized gas-conditioning protocol, not as a map normalized by measured dissolved-gas concentration.

This optical response is also controlled by bubble interior physics. SCL requires not only acoustic energy deposition but also radical production, excited-state chemistry, and transfer of chemically active species into the luminol-containing liquid. Gases that support intense collapse or oxygen-mediated chemiluminescent pathways therefore appear much brighter than gases that dissipate energy through stable oscillation or thermally leaky collapse. Argon can enhance SCL through strong collapse heating, oxygen and air through reactive oxygen chemistry and oxidant generation, and unsparged water through a broad native bubble population. Helium remains weaker because heat leakage reduces the chemical excitation efficiency, whereas CO_2_ and the boiled/headspace-free/vacuum-treated reference remain dark because radical-productive collapse is strongly suppressed. Thus, SCL provides a spatially resolved optical counterpart to the chemical decoupling already observed in the dosimetry results [3], [23].

The almost complete absence of SCL in the vacuum-treated gas-depleted reference is consistent with the combined boiling/headspace-free cooling/vacuum protocol, which was designed to minimize the dissolved-gas inventory before ultrasound exposure. This optical inactivity supports the use of the vacuum-treated liquid as a cavitation-suppressed reference, while still recognizing that the protocol does not prove absolute removal of every microscopic nucleus.

The first major point emerging from Fig. 8 is its clear decoupling from calorimetry. Calorimetry showed that unsparged water gave the highest total power in liquid, while oxygen, air, argon, and helium formed a relatively narrow upper group and carbon dioxide remained only slightly above the vacuum-treated reference. SCL sharpens this picture considerably. CO_2_-conditioned water and the vacuum-treated gas-depleted reference appear essentially dark, helium is visibly weaker than the other active gases, and oxygen, air, argon, and unsparged water show strong luminous cavitation zones. This means that the total thermal power measured calorimetrically is not sufficient to describe the emissive or chemically active fraction of the cavitation field. Within the ΔP-based energetic partitioning framework, the vacuum-treated gas-depleted reference defines the lower bound of the non-cavitational baseline and the excess over the vacuum-treated reference defines the apparent cavitation contribution. Fig. 8 then shows that this apparent cavitation contribution is not only chemically heterogeneous, as already demonstrated by dosimetry, but also spatially heterogeneous and optically heterogeneous. In other words, similar calorimetric increments can produce very different luminous cavitation patterns depending on dissolved gas composition [3], [20], [27].

The almost complete disappearance of emission in the vacuum-treated gas-depleted reference and CO_2_-conditioned water is especially significant. The vacuum-treated gas-depleted reference already showed a substantial calorimetric signal, about 14.6 W at 60 W and 24.1 W at 100 W, but the images reveal essentially no sonochemiluminescent activity. Carbon dioxide likewise gave a small but measurable calorimetric excess over the vacuum-treated reference, yet its images also remain essentially dark. This contrast confirms that most of the calorimetric signal under these conditions belongs to non-cavitational dissipation and not to chemically emissive cavitation. Such an observation strongly supports the ΔP interpretation adopted throughout the manuscript. The thermal signal that persists in the vacuum-treated gas-depleted reference is real, but it is not associated with a cavitation field capable of generating strong luminol emission, triiodide oxidation, ferric ion formation, hydrogen peroxide accumulation, aromatic hydroxylation, or effective dye degradation. The darkness of the vacuum-treated gas-depleted reference and CO_2_-conditioned water images is therefore not merely illustrative. It is one of the clearest visual validations of the distinction between non-cavitational dissipation and chemically productive cavitation [3], [20].

The relative brightness of oxygen, air, argon, and unsparged water also carries important mechanistic information. These four conditions all exhibit strong sonochemiluminescent activity, but the distribution and intensity of the bright regions are not strictly identical. Unsparged water and oxygen appear among the brightest conditions, with broad luminous zones occupying a large portion of the reactor volume. Air and argon also show strong emission, although the intensity distribution appears somewhat less homogeneous. Helium, by contrast, shows a weaker and more diffuse signal. This ranking is broadly compatible with the later chemical trends, but it does not reproduce any one dosimetry ranking exactly. That is expected. SCL is closely linked to cavitation collapse and reactive species generation, but it is still an optical probe with its own weighting toward emissive radical chemistry and local bubble conditions. Recent reviews and comparative studies have shown that sonoluminescence and SCL correlate positively with dosimetry, but the relationship is not one-to-one because different probes emphasize different subsets of bubble behavior and reaction chemistry [10], [14], [27].

The comparison with KI dosimetry is particularly informative. KI placed unsparged water at the top, followed by air and oxygen, whereas SCL visually suggests that unsparged water and oxygen are especially intense and helium is much weaker. This partial agreement indicates that both probes respond to cavitation-generated oxidizing activity, yet they do so through different mechanisms. KI measures integrated iodide oxidation, while SCL measures light emitted through luminol oxidation chemistry in the active cavitation zones. A condition may therefore sustain strong interfacial oxidation without giving exactly the same optical intensity ranking. The images nonetheless support the central conclusion from KI, namely that oxygen-containing and naturally equilibrated media convert the apparent cavitation contribution into much stronger chemically active cavitation than helium, CO_2_-conditioned water, or the vacuum-treated gas-depleted reference [10], [27]. The fact that CO_2_-conditioned water and the vacuum-treated gas-depleted reference are almost dark while unsparged water and oxygen are highly luminous is fully aligned with the KI trends and strongly reinforces the interpretation that the gas-dependent calorimetric increment is expressed as chemically active cavitation only under favorable gas-conditioned media [3], [10].

The comparison with KI-AHM further strengthens this view. The KI-AHM assay amplified oxygen-containing and unsparged conditions by capturing both direct oxidation and the peroxide-supported branch. The strong SCL observed in oxygen, air, and unsparged water is consistent with that behavior, because luminol emission also reflects a strongly oxidizing cavitation environment. However, SCL is more spatially immediate and visually discriminating than iodide chemistry. It shows not only that oxidizing chemistry occurs, but also where in the reactor it is most active. In this sense, Fig. 8 complements Fig. 3, Fig. 4 by demonstrating that the gas conditions producing the strongest combined oxidizing chemistry are also the conditions producing the brightest cavitation active zones. This is exactly the type of qualitative and spatial confirmation expected in a multimodal cavitation assessment [10], [27].

The relationship with Fricke and hydrogen peroxide dosimetries is even more revealing. Those two assays clearly favored oxygen, followed by air and argon, and therefore emphasized bulk oxidizing capacity and recombination chemistry. Fig. 8 supports the strong position of oxygen, because O_2_-conditioned water exhibits one of the brightest and most extensive luminous fields. Air also shows a strong emission pattern, consistent with its high Fricke and hydrogen peroxide responses. Argon shows marked emission as well, which indicates that it produces highly emissive and reactive collapse events even when it does not dominate the hydrogen peroxide ranking. Helium remains weaker, which is consistent with its lower Fricke and hydrogen peroxide responses. Thus, the SCL images occupy an intermediate interpretive position between the oxygen dominated bulk oxidant assays and the argon favored direct hydroxylation response measured later by 4-NP. They show that both oxygen-rich chemistry and intense localized collapse contribute to the observed optical activity [3], [14].

The comparison with 4-NP dosimetry is particularly useful because both probes are sensitive to chemically aggressive cavitation, yet they do not report the same outcome. The 4-NP data favored argon, which was interpreted as evidence that direct aromatic hydroxylation is especially promoted under the most energetic collapse conditions. Fig. 8 shows strong emission under argon, which supports that interpretation. However, oxygen and unsparged water also exhibit strong light emission, especially at 100 W, even though they do not all rank equally in the 4-NP assay. This means that SCL, while strongly related to chemically active collapse, is not restricted to the same direct aromatic hydroxylation pathway as 4-NP dosimetry. Instead, it reflects a broader luminol-responsive cavitation activity that includes highly emissive oxidizing events across the reactor. This again confirms that the apparent cavitation contribution defined by ΔP is partitioned among several chemical and optical branches rather than expressed through a single universal cavitation signature [10], [14], [27].

The power dependence visible in the images provides an additional energetic insight. For all active gases, the emission at 100 W is more intense and more spatially developed than at 60 W. This is especially evident for oxygen, air, argon, and unsparged water. Such a trend is consistent with the calorimetric observation that increasing electrical input increases both the total thermal signal and the apparent cavitation contribution. However, Fig. 8 makes clear that the additional energetic input is not merely dissipated as heat. Under favorable gas conditions, it is also converted into a more intense and more spatially extended luminous cavitation field. By contrast, the vacuum-treated gas-depleted reference and CO_2_-conditioned water remain essentially dark even at 100 W, which shows that increasing electrical power alone is not sufficient to generate chemically emissive cavitation if the dissolved gas state does not support it [3], [20]. In this sense, the SCL images visually validate the ΔP framework by showing that the gas-enabled calorimetric increment becomes optically and chemically meaningful only when the dissolved gas promotes active cavitation [3], [10].

Taken together, Fig. 8 confirms that SCL is a highly informative intermediate probe between calorimetry and chemical dosimetry. Calorimetry defines the total power dissipated in the liquid. The ΔP framework then isolates the gas-dependent apparent cavitation contribution. SCL shows where and under which gas conditions that apparent cavitation contribution is translated into optically emissive, chemically active cavitation. KI and KI-AHM reveal interfacial oxidation and peroxide-assisted oxidation, Fricke and hydrogen peroxide reveal broader bulk oxidizing capacity and recombination chemistry, 4-NP reveals direct aromatic hydroxylation, and Sunset Yellow FCF degradation ultimately integrates these branches into substrate destruction. Within this sequence, SCL serves as the crucial visual bridge that confirms the presence, distribution, and gas dependence of the active cavitation field before the final substrate-level outcome is considered [10], [14], [27].

Fig. 8 demonstrates that SCL provides a direct optical validation of the ΔP-based energetic partitioning, showing that the gas-dependent apparent cavitation contribution identified by calorimetry is translated into a bright and spatially extended active cavitation field only under favorable gas-conditioning states, particularly in oxygen, argon, air, and unsparged media, whereas the vacuum-treated gas-depleted reference and CO_2_-conditioned water remain largely non-emissive despite a measurable thermal signal.

### 3.6 Sunset Yellow FCF degradation

**Fig. 9:**
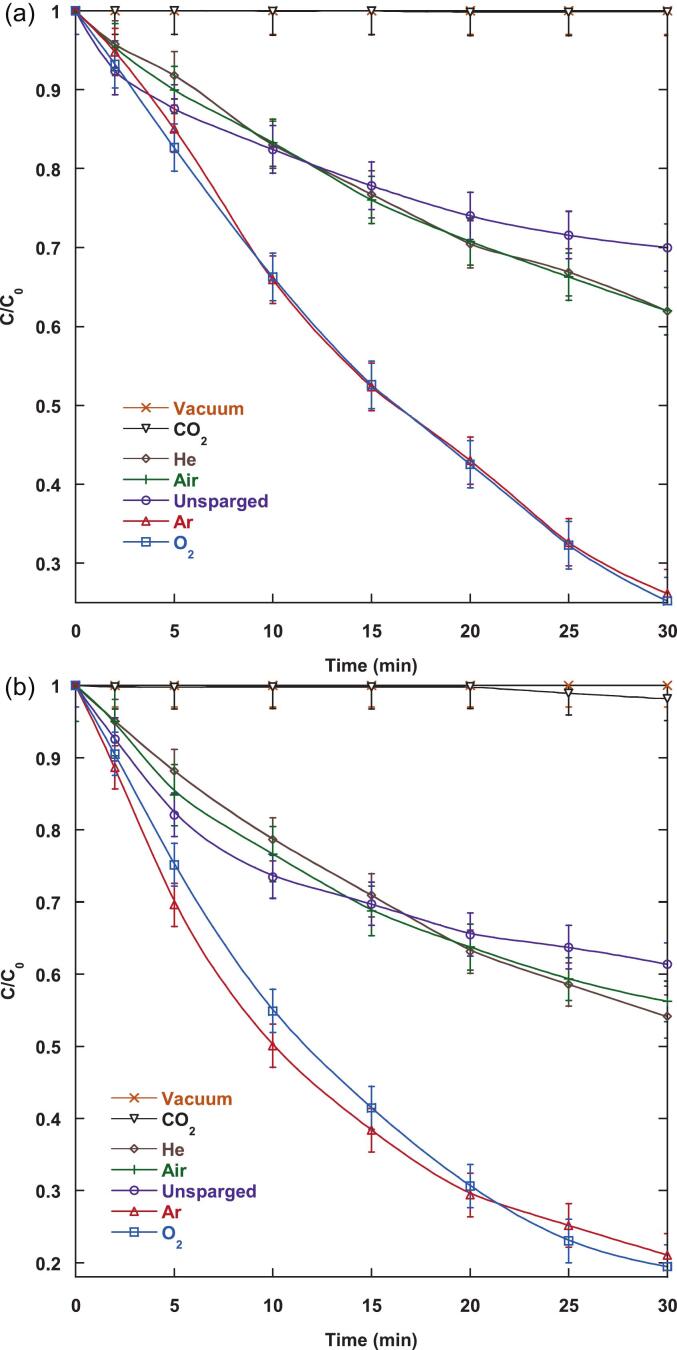
Sunset Yellow FCF degradation in the vacuum-treated gas-depleted reference, unsparged, and gas-conditioned media at (a) 60 and (b) 100 W (dye concentration: 5 mg/L, volume: 200 mL, frequency: 425 kHz, temperature: 20 °C).

Fig. 9a and 9b show that SSY degradation provides the most integrated chemical response of the entire gas-resolved framework. At 60 W, the normalized concentration after 30 min, C/C_0_, remained essentially unchanged in the vacuum-treated gas-depleted reference and CO_2_-conditioned water, with final values close to 1.00, whereas it decreased to about 0.63 in helium, 0.62 in air, 0.70 in unsparged water, 0.26 in argon, and 0.25 in O_2_-conditioned water. At 100 W, the final C/C_0_ values were again close to 1.00 in the vacuum-treated gas-depleted reference and CO_2_-conditioned water, but fell to about 0.54 in helium, 0.56 in air, 0.61 in unsparged water, 0.21 in argon, and 0.20 in oxygen. Expressed as degradation after 30 min, oxygen and argon therefore achieved roughly 74 to 75% removal at 60 W and about 79 to 80% removal at 100 W, while helium and air remained in the range of about 37 to 38% at 60 W and 44 to 46% at 100 W, and unsparged water reached only about 30% at 60 W and 39% at 100 W. These results immediately show that SSY degradation does not simply follow the total acoustic power dissipated in the liquid, but reflects the final chemical usefulness of the cavitation field after all energetic and chemical branches have been integrated into a substrate-specific response [3], [10].

The negligible SSY degradation under the vacuum-treated condition further supports the interpretation that the residual calorimetric signal measured after the combined boiling/headspace-free cooling/vacuum protocol is largely non-cavitational and chemically unproductive toward dye oxidation. Thus, P_cal,vac_ is not used as evidence for a completely bubble-free liquid, but as a conservative lower-bound thermal baseline for the ΔP analysis.

The first mechanistic conclusion emerges from comparison with calorimetry. The calorimetric data showed that unsparged water gave the highest total power in liquid, about 20.8 W at 60 W and 32.9 W at 100 W, while oxygen, helium, argon, and air formed a relatively narrow upper group. Fig. 9 does not reproduce that order. Oxygen and argon become the two leading conditions for dye destruction, whereas unsparged water, despite the highest calorimetric power, remains only intermediate. Within the ΔP-based energetic partitioning framework, the vacuum calorimetric signal defines the lower bound of the non-cavitational baseline, and the excess over the vacuum-treated reference defines the apparent cavitation contribution. The SSY curves then show that this apparent cavitation contribution is chemically heterogeneous. A condition may dissipate more total acoustic power and still degrade less dye if that power is not expressed in the oxidizing forms most relevant to destruction of the azo chromophore. This energetic and chemical decoupling is one of the principal reasons why calorimetry alone cannot predict sonochemical performance [3], [10].

The relationship with KI dosimetry is especially revealing. KI favored unsparged water, followed by air and oxygen, while argon remained below these conditions. SSY degradation shifts the ranking toward oxygen and argon. This difference shows that iodide oxidation and dye degradation are not governed by the same oxidative event. KI is highly sensitive to oxidation available to iodide near the bubble–liquid boundary, whereas SSY degradation requires that reactive species attack an organic chromophore and drive bond cleavage and progressive oxidation in a chemically productive manner. A condition such as unsparged water can therefore sustain strong interfacial oxidation without maximizing dye removal. Oxygen and argon appear to convert the apparent cavitation contribution into a more substrate-effective oxidative field than would be inferred from KI alone [10], [20].

The comparison with KI-AHM adds a second level of interpretation. The catalyzed iodide assay strongly amplified oxygen-containing and unsparged media because it captures both direct iodide oxidation and peroxide-assisted oxidation. Fig. 9 shows partial agreement with that pattern but not complete equivalence. Oxygen remains one of the best conditions for SSY degradation, which is consistent with a strong peroxide-supported oxidizing environment, yet unsparged water loses the leading position that it held in the iodide-based assays. This means that the larger oxidant inventory reflected by KI-AHM is clearly relevant to dye degradation, but it is not sufficient by itself to predict the final degradation ranking. SSY therefore behaves as a more demanding integrative probe than iodide, because it depends not only on oxidant abundance but also on the ability of those oxidants to attack and transform the dye efficiently [10].

The comparison with Fricke and hydrogen peroxide dosimetries is even more informative. Both Fricke and hydrogen peroxide placed oxygen at the top, followed by air and argon or argon and air, which indicates that oxygen-rich conditions maximize bulk oxidizing capacity and the recombination branch that generates hydrogen peroxide. Fig. 9 retains the strong position of oxygen, which confirms that these oxidative branches are highly relevant to SSY degradation. However, argon becomes nearly equivalent to oxygen in the degradation curves, especially at the end of the run, even though argon was not the highest condition in hydrogen peroxide formation. This means that dye degradation is not controlled solely by the accumulation of stable oxidants in the liquid. It also depends strongly on the subset of highly energetic collapse events that generate the most aggressive short-lived oxidizing species. Oxygen therefore contributes through a strong bulk oxidizing environment, while argon contributes through more severe collapse chemistry. SSY degradation benefits from both effects, which is why its ranking falls between the oxygen dominated pattern of Fricke and hydrogen peroxide and the argon favored pattern of 4-NP dosimetry [6], [10], [15].

The connection with 4-NP dosimetry confirms this interpretation. The 4-NP assay placed argon at the top and was interpreted as a selective indicator of direct aromatic hydroxylation under the most chemically aggressive collapse conditions. Fig. 9 partially follows that behavior because argon remains among the most effective conditions for SSY destruction. However, oxygen reaches essentially the same final conversion and even appears slightly superior at 100 W. This means that SSY degradation cannot be reduced to the same direct aromatic hydroxylation pathway measured by 4-NP alone. Instead, the dye appears to benefit from at least two complementary chemical advantages: the argon-type pathway associated with highly energetic radical attack, and the oxygen-type pathway associated with stronger bulk oxidizing capacity and peroxide-supported chemistry. The degradation curves therefore provide a chemically richer outcome than any single dosimetry method [3], [10].

From a bubble-physics perspective, SSY degradation integrates several of the pathways separated by the individual probes. Oxygen promotes a bulk oxidizing environment through oxygen-centered radicals, H_2_O_2_ accumulation, and peroxide-supported oxidation, whereas argon promotes severe collapse and short-lived radical attack near the interface. Both routes can contribute to chromophore destruction, bond cleavage, and progressive dye oxidation. This explains why SSY degradation converges toward oxygen and argon as the most effective conditions, even though oxygen dominates Fricke/H_2_O_2_ while argon dominates 4-NP. In contrast, unsparged water can dissipate the largest calorimetric power and show strong KI response without necessarily maximizing substrate-effective oxidation, because not all interfacial oxidation is equally effective for dye degradation. SSY therefore confirms that ΔP defines the available gas-enabled energetic increment, whereas the gas-specific bubble interior determines how much of that increment is converted into substrate-relevant oxidative chemistry [3], [23]. Because dissolved gas concentrations were not measured before and after SSY degradation runs, the degradation results are not interpreted in terms of absolute gas concentration or gas-consumption rates. They are interpreted as substrate-level outcomes of reproducible gas-conditioning protocols combined with the gas-specific cavitation chemistry resolved by the dosimetry and SCL measurements.

The vacuum-treated gas-depleted reference and CO_2_-conditioned water define the opposite end of the spectrum. Both conditions showed essentially no SSY degradation, even though calorimetry demonstrated a substantial thermal signal in the vacuum-treated gas-depleted reference and a small but measurable increase in CO_2_-conditioned water. This contrast confirms that most of the calorimetric signal present in the vacuum-treated gas-depleted reference belongs to non-cavitational dissipation and is chemically unproductive with respect to dye oxidation. Carbon dioxide behaves similarly, showing that even when a small apparent cavitation contribution is present, it may remain ineffective for substrate degradation if the gas suppresses radical-producing cavitation. This behavior is consistent with the known effect of carbon dioxide on bubble composition, oscillation damping, and collapse thermodynamics, all of which reduce the generation of the most chemically active cavitation events [3], [20].

The effect of increasing electrical power from 60 to 100 W provides an additional energetic insight. Oxygen and argon showed the strongest absolute increase in degradation performance, with the final residual fraction decreasing from about 0.25 to 0.20 in oxygen and from about 0.26 to 0.21 in argon. Helium, air, and unsparged water also improved, but remained much less reactive overall. This behavior indicates that increasing electrical input does not merely raise the total heat balance, as shown by calorimetry, but selectively enhances the fraction of the cavitation field that is able to oxidize the dye. In other words, the extra apparent cavitation power identified by ΔP analysis becomes practically meaningful only when the gas environment can convert it into substrate-effective chemistry. Gas composition and input power therefore act cooperatively in defining the final sonochemical outcome [3], [10].

A further consequence of Fig. 9 is that unsparged water, although highly effective in calorimetry and strong in iodide-based dosimetry, remains only an intermediate degradation condition. This is one of the most important findings of the study. Unsparged water evidently provides a favorable nuclei population and efficient overall energy coupling, which explains its strong calorimetric response. It also supports a substantial integrated oxidant output, which explains its high KI and KI-AHM responses. Yet the dye curves show that this condition does not maximize the final oxidative performance toward SSY. A reasonable interpretation is that unsparged water distributes the apparent cavitation contribution over a broader population of events that are energetically effective in aggregate but not optimized for the radical attack and oxidizing environment required for the fastest dye destruction. Oxygen and argon, by contrast, convert the same energetic framework into more substrate-effective chemistry [3], [10].

Taken together, Fig. 9 shows that SSY degradation is the final integrated expression of the gas-dependent cavitation physics and chemistry revealed by calorimetry and multidosimetry. Calorimetry defines the total power dissipated in the liquid. KI and KI-AHM define interfacial oxidation and peroxide-assisted iodide oxidation. Fricke and hydrogen peroxide define broader bulk oxidizing capacity and recombination products. 4-NP defines direct aromatic hydroxylation. SSY degradation then integrates the consequence of all these branches into one substrate-based performance metric. For this reason, Fig. 9 should not be expected to duplicate the ranking of any earlier assay exactly. Its scientific value lies precisely in the fact that it synthesizes the energetic and chemical information from the preceding figures into an application-relevant outcome [3], [10].

Fig. 9a and 9b demonstrate that SSY degradation is the most integrative readout of the gas-resolved framework, confirming that total dissipated power, interfacial oxidation, peroxide-supported oxidation, bulk oxidizing capacity, and direct aromatic hydroxylation all contribute to sonochemical performance, but with oxygen and argon providing the most effective overall conversion of the apparent cavitation contribution into dye destruction [3], [10].

### 3.7 **Energetic synthesis through** Δ**P-based partitioning**

Fig. 2 shows that the calorimetric data can be interpreted through a ΔP-based energetic partitioning framework in which the total power dissipated in the liquid is resolved into a non-cavitational baseline and an apparent cavitation-dependent contribution. Using the vacuum-treated gas-depleted reference as the cavitation-suppressed reference, the gas-enabled excess is expressed as ΔP = P_cal,gas_ − P_cal,vac_, while the total calorimetric signal is written operationally as P_total_ = P_non-cav_ + P_cav,app_, with P_non-cav_ ≈ P_cal,vac_ and P_cav,app_ ≈ ΔP.

The first conclusion from this analysis is that the non-cavitational baseline remains dominant under all conditions. At 60 W, the apparent cavitation contribution ranged from about 1.8 W in carbon dioxide to 6.2 W in unsparged water, while at 100 W it ranged from about 1.3 to 8.8 W. These values corresponded to only about 11.0 to 29.8% of the total calorimetric signal at 60 W and 5.1 to 26.7% at 100 W. Thus, most of the dissipated power remained associated with background thermal and hydrodynamic losses rather than chemically productive cavitation.

The second conclusion is that the apparent cavitation contribution is chemically heterogeneous. KI and KI-AHM showed that oxygen-containing and unsparged media converted this energetic increment efficiently into interfacial and peroxide-assisted oxidation. Fricke and H_2_O_2_ demonstrated that oxygen was most effective for bulk oxidizing capacity and peroxide accumulation. By contrast, 4-NP showed that argon was the most effective condition for direct aromatic hydroxylation, while SSY degradation identified oxygen and argon as the most favorable overall conditions for substrate destruction.

Taken together, the ΔP framework shows that calorimetry defines the energetic scale of the reactor, whereas the dosimetries and degradation experiments define the chemical destiny of the gas-dependent calorimetric excess. A larger ΔP therefore does not necessarily imply a higher sonochemical performance, because the same apparent cavitation contribution can be partitioned into different oxidative pathways depending on dissolved gas composition.

## 4 Conclusions

This work introduces a gas-resolved ΔP-based energetic partitioning framework for high-frequency sonoreactors that separates the total calorimetric signal into a non-cavitational baseline and an apparent cavitation contribution, then resolves the chemical expression of that contribution through multimodal cavitation assessment. The main innovation lies in coupling calorimetry with orthogonal chemical and optical probes to distinguish total power dissipation from chemically productive cavitation.

The divergence between ΔP and the individual dosimetric responses is a direct consequence of probe selectivity. ΔP quantifies the gas-enabled energetic excess relative to the cavitation-suppressed reference, but each chemical or optical probe samples a different outcome of bubble-collapse physics. KI emphasizes interfacial oxidants, KI-AHM captures peroxide-assisted oxidation, Fricke reflects a broader bulk oxidizing inventory, H_2_O_2_ reports radical recombination, 4-NP emphasizes aggressive near-interface hydroxylation, SCL maps chemically emissive cavitation zones, and SSY degradation integrates substrate-effective oxidative pathways. Therefore, gas identity governs not only how much acoustic energy becomes associated with cavitation, but also how that energy is chemically expressed.

Calorimetry showed that a substantial fraction of the total dissipated power persisted in the boiled/headspace-free/vacuum-treated gas-depleted reference, demonstrating the presence of a dominant non-cavitational contribution. The vacuum-treated reference was prepared by boiling ultrapure water, cooling it in a completely filled sealed vessel without headspace, and then applying vacuum treatment at < 1 kPa for 20 min immediately before ultrasonication. This protocol was designed to minimize dissolved gases and strongly suppress gas-assisted cavitation. However, because complete elimination of all microscopic nuclei cannot be proven, the vacuum-treated gas-depleted reference is best interpreted as a cavitation-suppressed or cavitation-minimized reference rather than an absolutely cavitation-free state. The gas-dependent calorimetric excess, expressed through the ΔP framework, is therefore an apparent cavitation contribution referenced to a deliberately gas-depleted baseline, not an absolute thermodynamic measure of cavitation power.

A further methodological clarification is that the dissolved concentrations of Ar, He, CO_2_, O_2_, and air components were not directly measured before or after ultrasonication. The gas variable in this work therefore represents a standardized conditioning protocol, not an independently quantified dissolved-gas concentration. Literature solubility and thermophysical properties are used only to rationalize the observed gas-dependent trends. This limitation does not affect the operational ΔP comparison, but it means that the reported responses should not be interpreted as concentration-normalized gas effects.

The dosimetric and degradation results further showed that this apparent cavitation contribution is chemically heterogeneous. KI and KI-AHM emphasized interfacial and peroxide-assisted oxidation, Fricke and H_2_O_2_ highlighted bulk oxidizing capacity, 4-NP identified argon as the most effective condition for direct aromatic hydroxylation, and SSY degradation showed that oxygen and argon gave the highest overall oxidative performance. Thus, the condition maximizing total dissipated power was not necessarily the condition maximizing sonochemical reactivity.

Overall, this work demonstrates that sonoreactor performance must be interpreted through a hierarchy of linked observables, from total dissipated power to pathway-specific chemistry and final substrate degradation. The proposed framework provides a more rigorous basis for reactor interpretation, gas selection, and process optimization than calorimetry or single dosimetry methods used in isolation.

## CRediT authorship contribution statement

**Oualid Hamdaoui:** Investigation, Conceptualization, Data curation, Methodology, Formal analysis, Resources, Validation, Project administration, Supervision, Funding acquisition, Visualization, Writing original draft, Writing – review & editing. **Abdulmajeed Baker:** Investigation, Visualization, Validation. **Lahssen El Blidi:** Methodology, Validation, Resources, Supervision. **Mohamed K. Hadj-Kali:** Validation, Visualization, Resources.

## Declaration of competing interest

The authors declare that they have no known competing financial interests or personal relationships that could have appeared to influence the work reported in this paper.
